# Safety Evaluation of Serendipity Berry Sweet Protein From *Komagataella phaffii*

**DOI:** 10.1002/jat.4781

**Published:** 2025-03-31

**Authors:** Yael Lifshitz, Shira Paz, Rotem Saban, Inbar Zuker, Hagay Shmuely, Katy Gorshkov, Jwar Meetro, Shahrzad Tafazoli, Trung Vo, Gabriela Amiram, Carmit Shani Levi, Uri Lesmes, Ilan Samish

**Affiliations:** ^1^ Amai Proteins Ltd. Rehovot Israel; ^2^ Intertek Health Sciences Inc. Mississauga Ontario Canada; ^3^ Laboratory of Chemistry of Foods and Bioactives, Department of Biotechnology and Food Engineering Technion–Israel Institute of Technology Haifa Israel

**Keywords:** digestibility, genotoxicity, monellin, protein, safety assessment, sweetener, toxicology

## Abstract

Serendipity Berry Sweet Protein (sweelin) is a novel hyper‐sweet thermophilic protein designed using Artificial Intelligence Computational Protein Design (AI‐CPD) to improve the stability and sensory profile of the protein found in serendipity berry ( *Dioscoreophyllum cumminsii* ). sweelin is produced through precision fermentation by expression in *Komagataella phaffii*. The safety of sweelin was investigated through an evaluation of its genotoxicity, mutagenicity, systemic toxicity and digestibility potential in *in vitro* and *in vivo* models. sweelin was not genotoxic in *in vitro* reverse mutation and mammalian micronucleus assays and was not associated with systemic toxicity in a 90‐day dietary toxicity study in rats. The no‐observed‐adverse‐effect level for sweelin in Sprague Dawley rats was established as 14,300 ppm, the highest dose tested. This dose level corresponds to dietary intakes of 838.3 and 946.0 mg/kg body weight/day in male and female rats, respectively. sweelin was demonstrated to be readily digestible in an *in vitro* semi‐dynamic model of the gastrointestinal tract. The results support the safety of sweelin as a food ingredient for sweetening purposes.

## Abstract 2

The safety of the novel sweet protein sweelin, produced via precision fermentation using *Komagataella phaffii*, was evaluated in a battery of genotoxicity studies, an *in vitro* digestibility study and a 90‐day dietary toxicity study. sweelin was not genotoxic and did not cause systemic toxicity in rats. The no‐observed‐adverse‐effect level for sweelin in Sprague Dawley rats was established as 14,300 ppm, the highest dose tested. sweelin was demonstrated to be readily digestible in an *in vitro* model of the gastrointestinal tract.

**Abbreviations**
AI‐CPD: Artificial Intelligence Computational Protein Design
ANOVA: analysis of variance
APTT: activated partial thromboplastin time
BAEE: N_α_‐benzoyl‐L‐arginine ethyl ester
Con A: Concanavalin A
CPBI: cytokinesis block proliferation index
DMSO: dimethyl sulphoxide
ELISA: enzyme‐linked immunosorbent assay
FOB: functional observational battery
GASE: General Assessment of Side Effects
GLP: Good Laboratory Practice
GRAS: Generally Recognized as Safe
IU: international units
ISO: International Organization for Standardization
K_2_EDTA: dipotassium ethylenediaminetetraacetic acid
LC–MS/MS: liquid chromatography with tandem mass spectrometry
MALDI‐TOF MS: matrix‐assisted laser desorption/ionization–time‐of‐flight mass spectrometry
MCHC: mean corpuscular haemoglobin
MNBN: micronucleated binucleate
MW: molecular weight
NOAEL: no‐observed‐adverse‐effect level
OECD: Organisation for Economic Co‐operation and Development
OGTT: oral glucose tolerance test
ppm: parts per million
PT: prothrombin time
RPM: revolutions per minute
RPMI: Roswell Park Memorial Institute
SBSP: serendipity berry sweet protein
SDS‐PAGE: sodium dodecyl sulphate–polyacrylamide gel electrophoresis
T3: triiodothyronine
T4: thyroxine
TSH: thyroid‐stimulating hormone

## 1 Introduction

Reducing sugar intake can significantly improve overall health and well‐being. Excessive sugar consumption has been associated with noncommunicable diseases such as diabetes, cardiovascular disease, metabolic syndrome, certain types of cancers and obesity (Huang et al. 2023; WHO 2023). In fact, 9.8% of Type II diabetes was demonstrated to be attributable to sugar‐sweetened beverages (Lara‐Castor et al. 2025). The World Health Organization (WHO) recommends reducing sugar intake to less than 10% of an individual's total daily energy intake as a preventative measure against these diseases (WHO 2015). As awareness of these health concerns grows, consumer demand for healthier alternatives that satisfy the desire for sweetness without compromising the taste of the food or health is increasing.

In foods and beverages, sugar plays a crucial role in enhancing taste through the activation of the sweet taste receptors on the tongue, sending signals to the brain's reward system and improving the overall palatability of the food (Gutierrez et al. 2020). Sweet proteins are a group of naturally occurring proteins that activate the sweet taste receptor T1R2/T1R3, leading to a sensation of sweetness. However, because of their proteinaceous nature, they are not metabolised by the human body in the same manner as other sugars and carbohydrate‐based sweeteners. Several sweet proteins from plants have been identified, including miraculin, monellin, thaumatin, mabinlin, pentadin, curculin, brazzein and neoculin (Agboola et al. 2014). Sweet proteins are significantly sweeter than sugar, offering an attractive alternative that would allow for reduction in the consumption of added sugar in the human diet. The sweet protein thaumatin has been approved for use as a sweetening agent in food and beverage products within the United States and at a global level by the Joint FAO/WHO Expert Committee on Food Additives for several decades. Brazzein, another sweet protein, produced by precision fermentation has Generally Recognized as Safe (GRAS) status for use as a sweetener (GRN 1142—U.S. FDA 2024); however, it is reported to be only 750‐fold sweeter than 5% sucrose solution.

Monellin is a sweet protein present in the serendipity berry from *Dioscoreophyllum cumminsii* , which grows in the West African region of Guinea, Democratic Republic of Congo, Nigeria and Mozambique (Kinghorn and Compadre 2012; Oselebe and Ene‐Obong 2007; Terashima and Ichikawa 2003). Serendipity berries have been consumed by the natives of Congo for their intense sweetening properties (Fry 2012; Inglett and May 1968; Morris and Cagan 1972; van der Wel 1972; van der Wel and Arvidson 1978). Wild‐type monellin consists of 94 amino acid residues and is composed of two intrinsically disordered chains: Chains A and B (Bohak and Li 1976). As a result, it forms a highly unstable heterodimer protein with a melting temperature of below 50°C, leading to its decreased protein stability (Kim et al. 1989). Furthermore, its unstable heterodimer structure affects its binding to the sweet taste receptor T1R2/T1R3, resulting in a sweetness potency of 1500 times that of sucrose on a weight basis (van der Wel 1972). Several research groups have attempted to redesign the monellin structure to improve its heat stability, resulting in synthesis of MNEI, a single‐chain form of monellin (Kim et al. 1989; Tancredi et al. 1992). Amai Proteins Ltd. (‘Amai Proteins’) has designed a novel sweet protein based on the single‐chain MNEI, called DM31, using Artificial Intelligence Computational Protein Design (AI‐CPD) (Samish 2017; Samish et al. 2011), where amino acid substitutions, additions and deletions have been made to improve biophysical and biochemical characteristics of the sweet protein, such as its thermostability, digestibility, sensory profile and pH stability. This was achieved by inserting protein sequence and structure motifs found in extremophilic proteins that reside in extreme conditions, thus enabling the protein to maintain functionality in the harsh conditions of food and beverage processing. Amai Proteins' Serendipity Berry Sweet Protein (hereafter referred to as ‘sweelin’), containing the sweet protein DM31, is of great interest to the food and beverage industry, as it offers a low‐calorie alternative to sugar and can be used in a variety of products including beverages, condiments, dairy, desserts and chewing gum. Amai Proteins' DM31 is approximately 3500 times sweeter than 6% sucrose water solution and enables up to 70% sugar reduction in numerous food applications without compromising the taste of the food or beverage products, as assessed by professional super‐taster sensory panels; thus, it presents an attractive option for individuals seeking to reduce their sugar intake.

Amai Proteins' sweelin, containing DM31, is obtained through precision fermentation of a genetically engineered strain of *Komagataella phaffii* (formerly known as *Pichia pastoris*) and follows standard fermentation processes that are closely controlled and monitored to ensure protein purity. To support the safety of Amai Proteins' sweelin for use as a food ingredient, a series of toxicological studies were conducted using standardised methods and guidelines developed by the Organisation for Economic Co‐operation and Development (OECD), along with a comprehensive semi‐dynamic *in vitro* digestion model to investigate the digestion profile of the protein. The toxicology studies included a bacterial reverse mutation test, an *in vitro* mammalian cell micronucleus test and a 90‐day repeated dose dietary toxicity study in rodents. The results of these studies, as described herein, support the safety of sweelin for use as a sweetening agent in food and beverage products.

## 2 Materials and Methods

### 2.1 Test Material

sweelin is characterised by a sweet protein called DM31, which is a single‐chain protein comprised of 93 amino acids. DM31 is 92% identical to the single‐chain monellin, MNEI, and 94% identical to the wild‐type monellin found in serendipity berry. The amino acid sequence of DM31 is as follows:

GNWEIIDIGPFTQNLGKFAVDEANKIGQYGRLTFNKVIRPCMKKTIYENGEIKGYEYQLYVRASDKIFRADISEDYKTRGRKLLRFNGPVPPP

**FIGURE 1:**
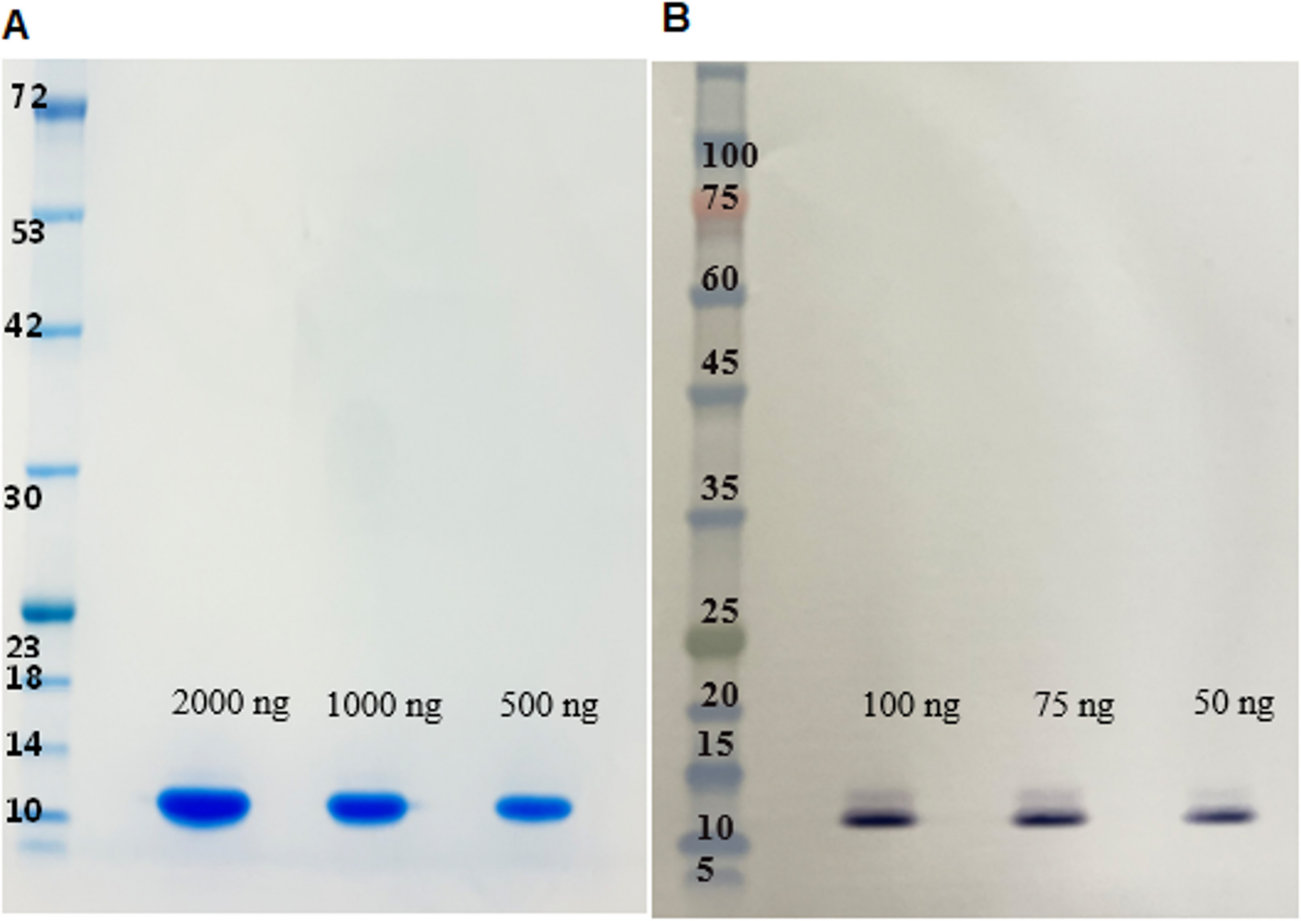
Identification of DM31 protein presence in sweelin. sweelin final product was separated on 12% bis‐tris acrylamide SDS‐PAGE. The loaded protein amounts are indicated in the figure. (A) Coomassie Blue staining was performed to assess purification levels and determine the molecular size of DM31. (B) Western blot analysis identified DM31 using a polyclonal antibody. Alt text: SDS‐PAGE gel demonstrating the molecular weight of sweelin using different methods.

The protein has a calculated molecular weight (MW) of 10.8 kDa based on its amino acid sequence and confirmed by sodium dodecyl sulphate–polyacrylamide gel electrophoresis (SDS‐PAGE) with Coomassie blue staining and by Western blot applying a polyclonal antibody (Figure 1). The MW of DM31 was confirmed to be 10.8 kDa by matrix‐assisted laser desorption/ionization–time‐of‐flight mass spectrometry (MALDI‐TOF MS, Ben Gurion University of the Negev, Beer Sheva, Israel) analysis.

Over the past 4 years, Amai Proteins' professional sensory panel has assessed sweelin biweekly, with 12–16 tasters per session, drawn from a pool of 20–30 experts trained and calibrated in analytical discrimination and selected according to the ISO 8586 standard. This panel was used to rate the sweetness intensity of sweelin that was demonstrated to be 3500 times sweeter than 6% sucrose water solution.

In the 14‐day dietary and 90‐day dietary toxicology studies conducted in rats, the sweet protein DM31 constituted 80% and 86% of the sweelin powder, respectively. In the genotoxicity studies, the DM31 content in the sweelin powder was 68.5%. The sweelin powder is absent of chemical and microbiological contaminants, heavy metals and residual DNA from the production strain.

### 2.2 Sensory Time–Intensity

The time–intensity of sweelin compared with sucrose was determined by the super‐taster sensory panel. The time–intensity evaluation is defined as a dynamic sensory analysis to measure the sweetness intensity over time in response to a single administration of the test item (Bi 2015). The panel was precalibrated on a 0‐to‐100 scale using a sucrose calibration system, which corresponded to 2%, 4%, 6% and 8% sucrose water solutions, representing 25, 50, 75 and 100 on the scale, respectively.

### 2.3 Investigation of *In Vitro* Digestibility

The digestibility of sweelin was investigated using a computer controlled *in vitro* semi‐dynamic digestion model based on INFOGEST (Brodkorb et al. 2019; Shani‐Levi et al. 2013). A dual auto titration unit (Titrando 902, Metrohm, Switzerland) was used to mimic physiological conditions of healthy adult gastric and duodenal digestion; it comprised a 100‐mL double‐jacket vessel controlled by a preprogrammed plan (TIAMO 2.5 software, Metrom) to mimic the pH gradient in the human stomach followed by an intestine phase of static pH (Shani‐Levi et al. 2013). The temperature was set to 37°C and stirring was set to 250 RPM. Protease enzymes (listed below), bile salts (sodium glycodeoxycholate and taurocholic acid sodium salt hydrate) and simulated digestive fluids (saliva, gastric and duodenal) were set according to the INFOGEST protocol (Brodkorb et al. 2019). No enzymes were added to the oral phase; pepsin (≥ 3200 U/mg) was added in the gastric phase; and trypsin (13,000–20,000 BAEE U/mg) and α‐chymotrypsin (≥ 40 U/mg) were added to the duodenal phase. The proteolysis was performed using ratios of 1:1 and 1:10 of sweelin (total protein):pepsin in physiological conditions.

**FIGURE 2:**
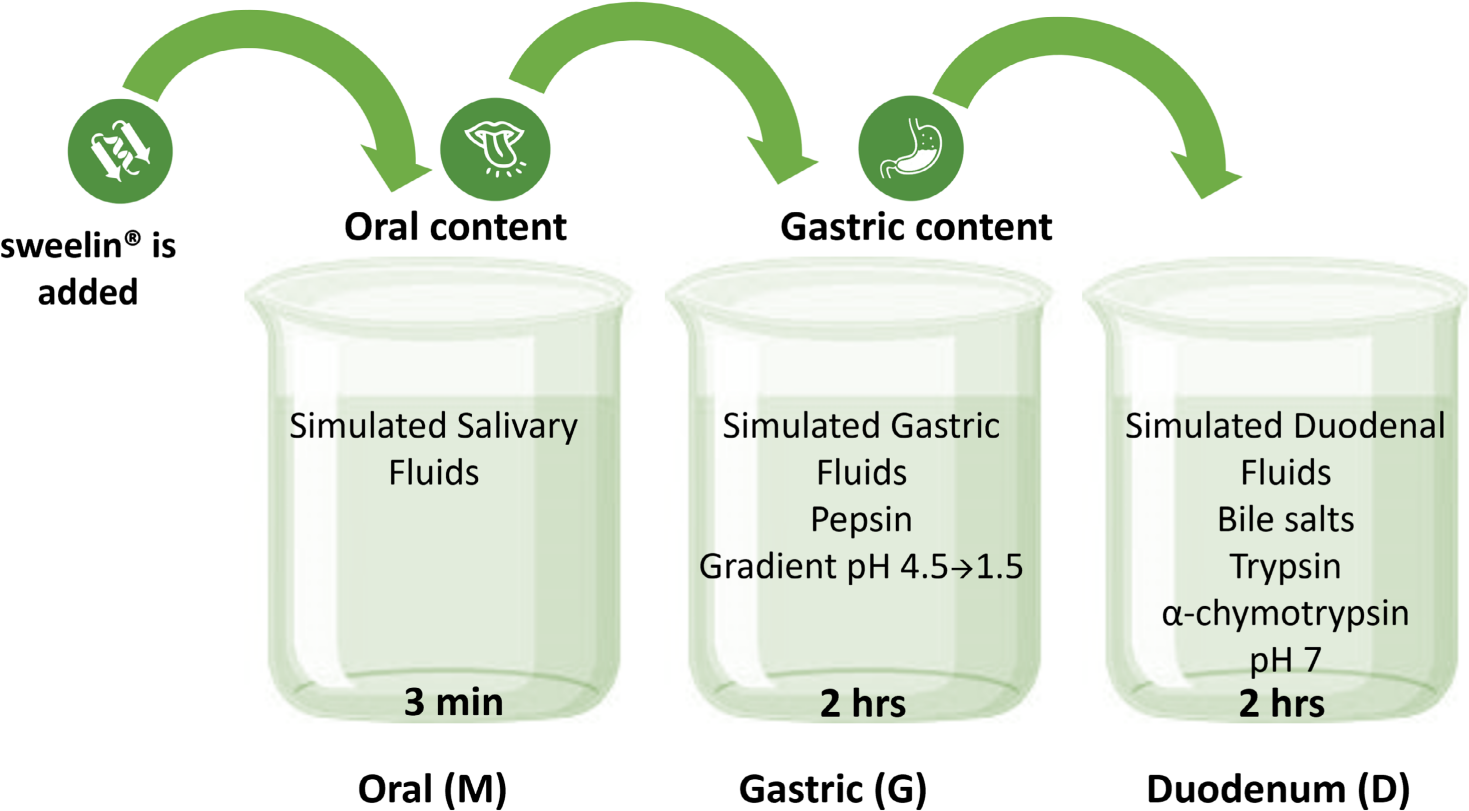
Schematic of the phases of the human digestibility experiments. Alt text: Illustration of the phases of the human digestibility experiments.

The digestibility assays tested 20 or 100 mg of sweelin dissolved in 25 mL of 5‐mM sodium citrate buffer at pH 6.0. Three digestibility phases were evaluated—oral, gastric and duodenal—for 3 min, 2 h and 2 h, respectively, as described in Figure 2. During and at the end of each phase, digesta samples were collected at different timepoints. In the gastric phase, samples were collected after 5, 15, 30, 60 and 120 min. In the duodenal phase, samples were collected after 5, 10 or 15, 20, 60 and 120 min. Collected digesta samples were analysed by SDS‐PAGE with Coomassie blue staining or Western blotting. Bioaccessible peptides were also analysed by Tricine–SDS‐PAGE with Coomassie blue staining and by liquid chromatography with tandem mass spectrometry (LC–MS/MS, Technion, Haifa, Israel).

### 2.4 Genotoxicity Studies

#### 2.4.1 Bacterial Reverse Mutation Test

The bacterial reverse mutation test (Ames test) was conducted at Labcorp Early Development Laboratories Ltd. (Harrogate, UK) in accordance with the OECD Test Guideline 471 (*Bacterial Reverse Mutation Test*—OECD 1997) and Good Laboratory Practice (GLP) (OECD 1998a). *Salmonella typhimurium* strains TA98, TA100, TA1535, TA1537 and TA102 were evaluated in the presence and absence of metabolic activation. All strains were originally obtained from Molecular Toxicology Incorporated, Boone, NC, USA, with the exception of TA102, which was obtained from Labcorp Early Development Laboratories Ltd. (Harrogate).

The main test was conducted with and without metabolic activation by the plate incorporation method. Seven dose levels (5, 16, 50, 160, 500, 1600 and 5000 μg/plate) were tested in triplicate. Water (0.1 mL) was used as the vehicle control. The positive controls included sodium azide, 9‐aminoacridine, 2‐nitrofluroene, 2‐aminoanthracene, benzo(a)pyrene, cumene hydroperoxide and 1,8‐dihydroxyanthraquinone (danthron). All positive controls were obtained from Sigma‐Aldrich (St. Louis, MO, United States), with the exception of danthron, which was obtained from Thermo Fisher Scientific (Waltham, MA, United States), and were formulated using dimethyl sulphoxide (DMSO). The mammalian liver post‐mitochondrial fraction (S9) used for metabolic activation was obtained from Molecular Toxicology Incorporated, where it was prepared from male Sprague Dawley rats (Charles River Laboratories Inc., Raleigh, NC, USA) induced with β‐naphthoflavone/phenobarbital. The S9 was supplied as lyophilised S9 mix (Mutazyme [Molecular Toxicology Incorporated]).

The platings were achieved by the following additions to 2 mL‐supplemented molten top agar: 0.1‐mL bacterial culture; 0.1‐mL test article solution, vehicle or positive control; 0.5‐mL 10% S9 mix; or buffer solution. This was followed by rapid mixing and pouring on to Vogel‐Bonner E agar plates. When set, the plates were inverted and incubated protected from light for 3 days in an incubator at 34°C–39°C. Following incubation, the plates were examined for evidence of toxicity to the background lawn, and where possible, revertant colonies were counted.

A confirmatory test using the pre‐incubation method was also performed. Quantities of test article, vehicle control solution or positive control, bacteria and S9 mix or buffer solution, as detailed above, were mixed and placed in an orbital incubator at 34°C–39°C for 20 min before the addition of 2‐mL molten agar. Plating of these treatments then proceeded using the plate‐incorporation procedure described above.

For both the main and confirmatory tests, revertant colonies were counted electronically using a Sorcerer Colony Counter (Perceptive Instruments [part of the Instem Group, Stone, Staffordshire, UK]). The toxicity of the test material was evaluated for abnormalities based on evaluation of the background bacterial lawn. A test was considered positive if (i) treatments provided a concentration‐related increase in revertant numbers at one or more concentrations in at least one strain with or without metabolic activation system; (ii) the increase in mean revertant colony numbers per plate was ≥ 1.5‐fold (in strain TA102), ≥ 2‐fold (in strains TA98, TA100) or ≥ 3‐fold (in strains TA1535 or TA1537) the concurrent vehicle control values; and (iii) any increase was reproducible, where applicable. A test was considered negative if all three criteria were not met.

#### 2.4.2 *In Vitro* Mammalian Micronucleus Assay

An *in vitro* micronucleus assay was conducted at Labcorp Early Development Laboratories Ltd. (Harrogate) in accordance with previously described methods (Aardema et al. 1998; Elhajouji et al. 1998; Fenech 1998; Fenech et al. 1999; Fenech et al. 2003; Migliore and Nieri 1991; Miller et al. 1998; OECD 2023; Rosefort et al. 2004; Thybaud et al. 2007). Human peripheral blood lymphocytes from two healthy, non‐smoking donors were obtained via venous puncture and collected in heparinized tubes. Whole blood cultures were established by placing 0.4 mL of pooled blood and 0.2 mL of phytohaemagglutinin into 8.3 mL of HML media (Roswell Park Memorial Institute [RPMI] medium [Sigma‐Aldrich] containing 10% [v/v] heat‐inactivated foetal bovine serum, 0.2‐IU/mL sodium heparin, 20‐IU/mL penicillin/20 μg/mL streptomycin and 2.0‐mM L‐glutamine) so that the final volume following addition of S9 mix/potassium chloride and the test article in the chosen vehicle was 10 mL. All cultures were then incubated at 34°C–39°C, and the cells were resuspended (twice daily) by gentle inversion. Cultures were incubated for approximately 48 h prior to treatments.

The S9 fraction used for metabolic activation was obtained from Molecular Toxicology Incorporated, where it was prepared from male Sprague Dawley rats induced with β‐naphthoflavone/phenobarbital. The S9 was supplied as lyophilised S9 mix (Mutazyme).

An initial analysis for test article cytotoxicity was conducted with and without S9 metabolic activation. Tests were conducted using 3.91, 7.81, 15.63, 31.25, 62.5, 125, 250, 500, 1000 and 2000 μg/mL of sweelin. Cytostasis was assessed via cytokinesis block proliferation index (CBPI) using the formula:

$$\text{Cytostasis}=100-\left(\left({\text{CBPI}}_{T}-1/{\text{CBPI}}_{C}-1\right)\right)$$

where CBPI_T_ was for the test and CBPI_C_ was for the controls.

CBPI was calculated as follows:

$$\begin{array}{c} \text{CBPI}=[\left(\text{mononucleate cells}\right)+\left(\text{binucleate cells}\times 2\right)\\ \;+\left(\text{multinucleate cells}\times 3\right)]/\text{Total number of cells} \end{array}$$

For the short‐term exposure experiments, duplicate cultures were tested at 500, 1000 and 2000 μg/mL of the test article with and without S9 activation. Vehicle control cultures were tested as four replicates. Cultures were exposed to the test article for 3 h, after which cells were pelleted (approximately 500 g, 5 min), washed once with sterile saline (prewarmed in an incubator at 34°C–39°C) and resuspended in fresh prewarmed HML media. The cultures were then incubated for an additional 17 h. Cultures from each dose group were scored.

For the long‐term exposure experiment, duplicate cultures (four replicates for the controls) were tested at the same concentrations. Cyto‐B formulated in DMSO was added at the initiation of treatment. Cultures were exposed to sweelin for 20 h, and cultures from all doses were scored for micronuclei.

At the defined sampling time, cultures were centrifuged at approximately 500 g for 5 min, the supernatant was removed and discarded, and cells were resuspended in 4 mL (hypotonic) 0.075 M potassium chloride at 34°C–39°C for 3 min to allow for cell swelling. The cultures were then agitated, and the cells were fixed by adding 4 mL fresh, cold methanol/glacial acetic acid (3:1, v/v) slowly onto the culture surface and slowly inverting to mix. The cultures were centrifuged at approximately 500 g for 5 min. The supernatant was removed, and the cell pellet was resuspended in the residual supernatant. An additional 4 mL of fresh fixative was then added, and the cells were stored at 2°C–8°C for a minimum of 30 min until slide preparation. For slide preparation, cells were centrifuged (approximately 500 g, 5 min), the supernatant was removed, and the cell pellet was resuspended in the residual supernatant to yield a milky suspension. Approximately 50 μL of cell suspension was gently spread onto clean, dry microscope slides. Slides were stained by immersion in 12.5 μg/mL Acridine orange in purified water for approximately 4 min, followed by immersion in purified water for 5 min then immersion in tap water for 2 min. Scoring was carried out using fluorescence microscopy. A minimum of 1000 binucleate cells from each culture (2000 per test concentration; 4000 for the vehicle control group) were analysed for micronuclei. The number of cells containing micronuclei on each slide was recorded.

The test was considered to be clearly positive if all of the following criteria were met: (i) a statistically significant increase in the frequency of micronucleated binucleate (MNBN) cells at one or more concentrations was observed; (ii) an incidence of MNBN cells at such a concentration that exceeded the normal range in both replicates was observed; and (iii) a concentration‐related increase in the proportion of MNBN cells was observed (positive trend test).

### 2.5 Repeated Dose Oral Toxicity Studies in Rodents

#### 2.5.1 14‐Day Dietary Study

A 14‐day dietary study was conducted to evaluate the palatability and general toxicity of sweelin in the diet and to determine the appropriate doses to be used in a subsequent longer subchronic study. The study was conducted at Product Safety Labs (Dayton, New Jersey, USA) and its design was based on OECD Test Guideline 407 (*Repeated Dose 28‐day Oral Toxicity Study in Rodents*—OECD 1995) and the *U.S. FDA Redbook 2000 IV.C.4* (U.S. FDA 2003). The study was conducted in a GLP‐compliant facility.

Adult CRL Sprague Dawley CD IGS rats (*n* = 5/sex/group, 8 weeks old) supplied by Charles River Laboratories (Wilmington, MA, USA) were provided for targeted daily intakes of 250, 500 or 1100‐mg/kg body weight/day of sweelin powder. The dose levels were selected based on a daily feed consumption of 25 g for a 300‐g rat, providing diets containing 3000, 6000 or 13,200 ppm sweelin powder. The commercial feed used in this study was the 2016 Certified Teklad Global Rodent Diet with 16% protein, obtained from Inotiv Teklad Inc. (West Lafayette, IN, USA). Animals in the control group received the feed with no test article added. Diets and water were available *ad libitum*. The stability, homogeneity and achieved concentrations of sweelin in each test diet were confirmed. Animals were individually housed in steel cages and acclimated with 12‐h light/dark cycles for 6 days prior to the dosing period. The temperature and relative humidity of the room was 22°C and 32%–40%, respectively, for the duration of the study period.

The animals were observed daily for viability, signs of gross toxicity and behavioural changes. Detailed clinical observations were conducted weekly while handling the animals. Body weights were recorded twice during the acclimation period (including prior to initial dietary administration on Day 0), as well as on Days 3, 7, 10 and 14. Body weight gain was calculated for selected intervals and for the entire study duration. Individual food consumption was recorded on days coinciding with body weight measurements. Food efficiency and dietary intake of sweelin were calculated. At study termination, a gross necropsy was performed on all animals without fasting.

Statistical analyses were conducted using Prism Biostatistics (GraphPad Software, San Diego, CA, USA). Body weight parameters, food consumption and food efficiency were analysed using a two‐way analysis of variance (ANOVA). Significant interactions observed between treatment and time, as well as main effects and non‐significant findings, were further analysed by a post hoc multiple comparisons test (e.g., Dunnett's test) of the individual treated groups to the control group.

#### 2.5.2 90‐Day Dietary Study

The 90‐day dietary study in rats, which followed OECD Test Guideline 408 (*Repeated Dose 90‐Day Oral Toxicity Study in Rodents*—OECD 1998b), was conducted at Product Safety Labs (New Jersey), using 80 CRL Sprague Dawley CD IGS rats (*n* = 10/sex/group, 8 weeks of age) supplied by Charles Rivers Laboratories Inc. Animals were individually housed in steel cages conforming to the size recommendations in the latest *Guide for the Care and Use of Laboratory Animals* (NRC 2011) and acclimated with 12‐h light/dark cycles for 6 days prior to the dosing period. The temperature and relative humidity of the room ranged from 21°C to 24°C and 46%–61%, respectively, for the duration of the study period. Animals were stratified by body weight and assigned to groups by randomisation to avoid any significant differences in body weight in any test group. Although the rats used in the study were considered to be pathogen‐free, surveillance for study animals was carried out by using two male rats and one female rat as ‘sentinel’ animals that were not part of the 91‐day study. Sentinel animals were clearly marked and housed under the same conditions alongside the study animals for 91 days. Serum samples were collected from sentinel animals for screening of common rat pathogens (Rat Parvovirus, Toolan's H‐1 Virus, Kilham Rat Virus, Rat Minute Virus, Parvovirus NS‐1, Rat Coronavirus, Rat Theilovirus and *Pneumocystis carinii*). The serum samples were sent on ice to IDEXX BioAnalytics (Columbia, MO, USA) for evaluation. Based on the negative results for all pathogens, the study animals were considered to be healthy and free of the tested pathogens.

Animals were provided with diets prepared containing targeted daily intakes of 250 (low dose), 500 (mid dose) and 1100 mg/kg body weight/day (high dose) of sweelin powder. The dose levels were selected based on a daily feed consumption of 27 g for a 350‐g rat, providing dietary concentrations of 3250, 6500 and 14,300 ppm sweelin powder (equivalent to 0.325%, 0.650% and 1.430%). At the initiation of the dosing period, males weighed 217–265 g and females weighed 182–217 g. The same feed used in the 14‐day study was used for the 91‐day study. The test diets containing the feed and test article were available *ad libitum* for the 91‐day dosing period, and filtered tap water was also available *ad libitum*. sweelin was added to the feed and mixed thoroughly in a high‐speed metal mixing bowl with a paddle for at least 20 min. The control diet was mixed under the same conditions without the addition of the test article. All diets were prepared weekly and refrigerated unless they were provided to the animals on the same day. The stability of the test article was confirmed using frozen samples collected at the beginning (Day 0), middle (Week 7) and end of the in‐life phase (Week 13). The stability of the test article within the dietary matrix was confirmed using samples from the highest and lowest test diet concentrations. The homogeneity of the test diets was confirmed by collecting samples from the top, middle and bottom strata of the dietary preparations at the lowest and highest concentrations. The achieved concentration of sweelin in each test diet was also confirmed in samples collected from the beginning, middle and end of the study period.

Cage‐side clinical observations were performed daily, and animals were observed for mortality at least twice daily. Body weights and feed consumption were recorded during the acclimation period, on Day 0 and weekly thereafter. Detailed observations were conducted weekly while handling the animals, generally coinciding with the assessment of feed consumption and body weights. Animals were observed for their appearance, posture, behaviour, excretions and autonomic activity (e.g., lacrimation, piloerection, pupil size or unusual respiratory patterns). Ophthalmologic evaluations were conducted for all animals prior to study initiation and on Day 81.

Tests for functional observational battery (FOB) were conducted at the end of the study period on Day 80. Animals were evaluated in an open field for excitability, autonomic function, gait, sensorimotor coordination, reactivity, sensitivity (elicited behaviour) and other abnormal clinical signs (e.g., convulsions, tremors, unusual or bizarre behaviour, emaciation, dehydration and general appearance). In addition, forelimb and hindlimb grip strength and foot splay measurements were recorded. Motor activity of each animal was evaluated for a 1‐h phase on Day 81 using an automated Photobeam Activity System (San Diego Instruments, Inc, San Diego, CA, USA).

Blood samples were collected from all animals on the day of necropsy (Day 92 for males and Day 93 for females) for evaluation of haematology, clinical chemistry and coagulation parameters, as well as urinalysis and thyroid hormone analysis. Animals were fasted overnight, and blood samples were collected sublingually and/or from the vena cava under isoflurane anaesthesia. For haematological evaluation, approximately 500 μL of blood was collected from each animal in a precalibrated tube containing dipotassium ethylenediaminetetraacetic acid (K_2_EDTA) and refrigerated until analysis. For blood coagulation analysis, 1.8‐mL blood samples were collected in precalibrated tubes containing 3.2% sodium citrate. The samples were centrifuged in a refrigerated centrifuge, and the plasma was transferred into labelled tubes. Plasma samples were stored at −80°C until the analysis of activated partial thromboplastin time (APTT) and prothrombin time (PT). For clinical chemistry evaluation, 1000‐μL blood samples were collected into tubes with no preservative and were processed in a refrigerated centrifuge to separate the serum. Serum samples were stored in labelled tubes at −80°C until analysis. These serum samples were also analysed for triiodothyronine (T3), thyroxine (T4) and thyroid‐stimulating hormone (TSH) using an enzyme‐linked immunosorbent assay (ELISA) method. Urine samples were collected from fasted animals on Day 92 and Day 93 for males and females, respectively, and stored on ice until urinalysis was conducted.

Scheduled termination and necropsy occurred on Day 92 for males and Day 93 for females. All animals were euthanised by exsanguination under isoflurane anaesthesia and subject to gross examination of the external surface of the body, all orifices, the musculoskeletal system and the cranial, thoracic, abdominal and pelvic cavities. The following organs were weighed as soon as possible after dissection to determine the absolute and relative organ weights: adrenals (combined), brain, epididymis (combined), heart, kidneys, liver, ovaries with oviduct (combined), spleen, testes (combined), thymus and uterus. These organs were also preserved in 10% neutral buffered formalin for histopathological examination. Additional tissues that were preserved in 10% neutral buffered formalin and weighed after at least 24 h of preservation included prostate and seminal vesicles with coagulating gland (combined), thyroid and parathyroid and the pituitary gland.

Histopathological examinations were performed on the preserved organs and tissues of animals from the control group and the high‐dose group. The fixed tissues were trimmed and embedded in paraffin, sectioned with a microtome, placed on glass microscope slides and stained with haematoxylin and eosin prior to examination by light microscopy.

Statistical analyses for all in‐life endpoints identified as multiple measurements of continuous data over time (e.g., body weight parameters, food consumption, food efficiency and FOB endpoints) in treatment and control groups were compared using a two‐way ANOVA, testing the effects of both time and treatment. Significant interactions observed between treatment and time, as well as main effects and non‐significant findings, were further analysed by a post hoc multiple‐comparison test (e.g., Dunnett's test) of the individual treated groups to the control group. Organ weights and clinical pathology results (haematology, clinical chemistry and urinalysis) were evaluated for homogeneity of variances (Bartlett's test) and normality (Shapiro–Wilk test). Where homogeneous variances and normal distributions were observed, treatment and control groups were compared using one‐way ANOVA. When the ANOVA indicated a significant difference, a comparison of the treated groups to control was performed with a multiple comparisons test (e.g., Dunnett's test). Where variances were considered significantly different, groups were compared using a non‐parametric method (e.g., Kruskal–Wallis non‐parametric ANOVA) and where significant effects were observed, comparisons of treated groups to control were performed (e.g., Dunn's test). All analyses were conducted using Provantis version 10, Tables and Statistics (Instem LSS, Staffordshire, UK) and Prism Biostatistics (GraphPad Software, San Diego, CA, USA).

## 3 Results

### 3.1 Sensory Time–Intensity

**FIGURE 3:**
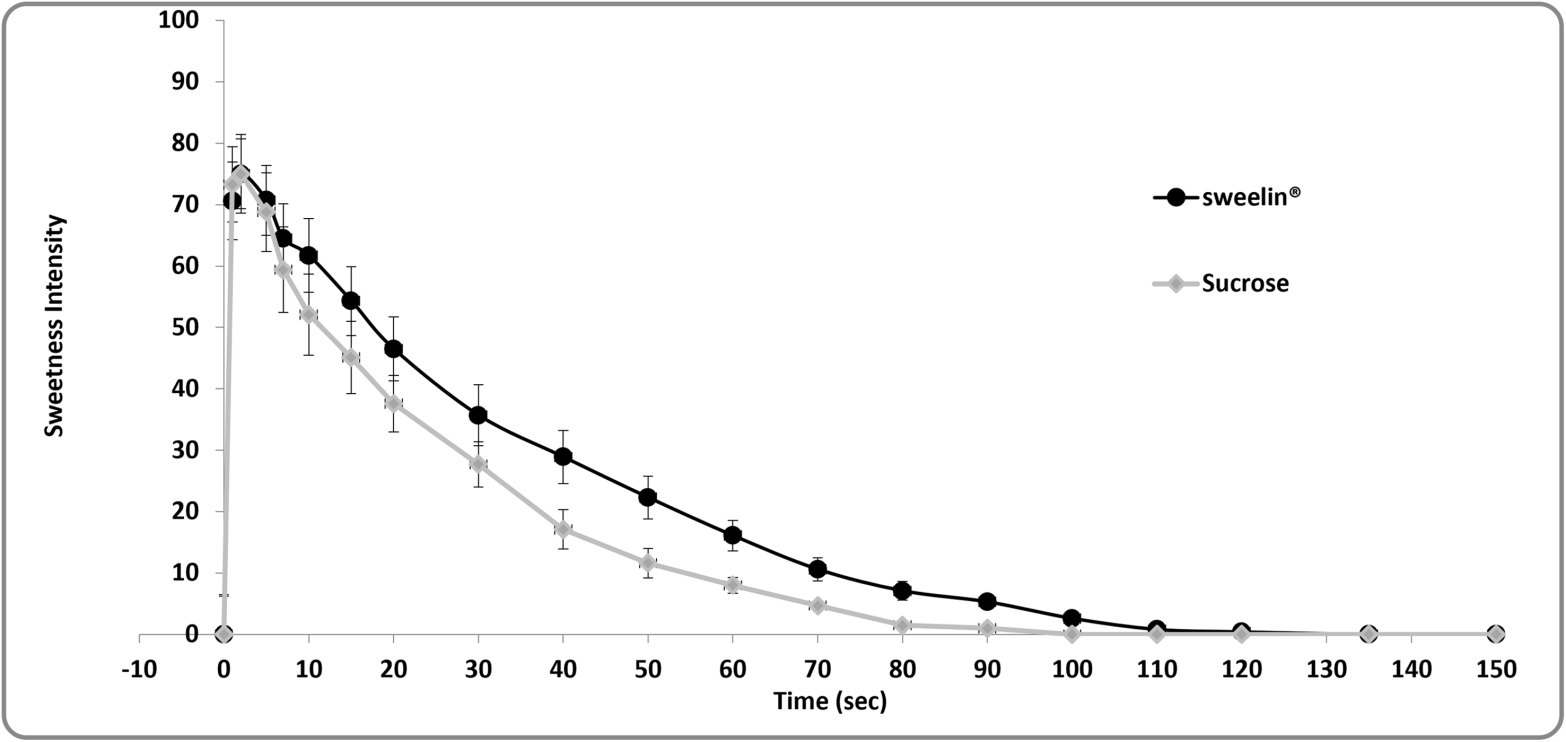
Sweetness intensity of sweelin reduces over time in similar manner as sucrose. Time intensity method rated by sensory expert panel that was precalibrated on a 0‐to‐100 scale using a sucrose calibration system, which corresponds to 2%, 4%, 6% and 8% sucrose water solutions, representing 25, 50, 75 and 100 on the scale, respectively. The graph presents mean ratings of sweetness intensity of sweelin and sucrose, with their standard errors represented by error bars. Alt text: Graph representing the sweetness intensity of sweelin in comparison to sucrose.

The sweetness intensity of sucrose reached 0 on the sweetness intensity scale within 100 seconds of administration (Figure 3). In comparison, sweelin reached a sweetness intensity of 0 at 135 seconds. Although the decline rate for sweelin was comparable, it was slightly slower than that of sucrose.

### 3.2 *In Vitro* Digestibility

sweelin was readily digestible under intestinal conditions where bile and pancreatic secretions were added. In fact, the presence of physiological levels of trypsin and α‐chymotrypsin led to significant degradation of sweelin. LC–MS/MS analysis of bioaccessible peptides reaffirmed the observations that sweelin is readily digested in the intestine into very short peptides. After 2 h of intestinal digestion, undigested protein levels were estimated to be less than 3% of the total ingested dose.

**FIGURE 4:**
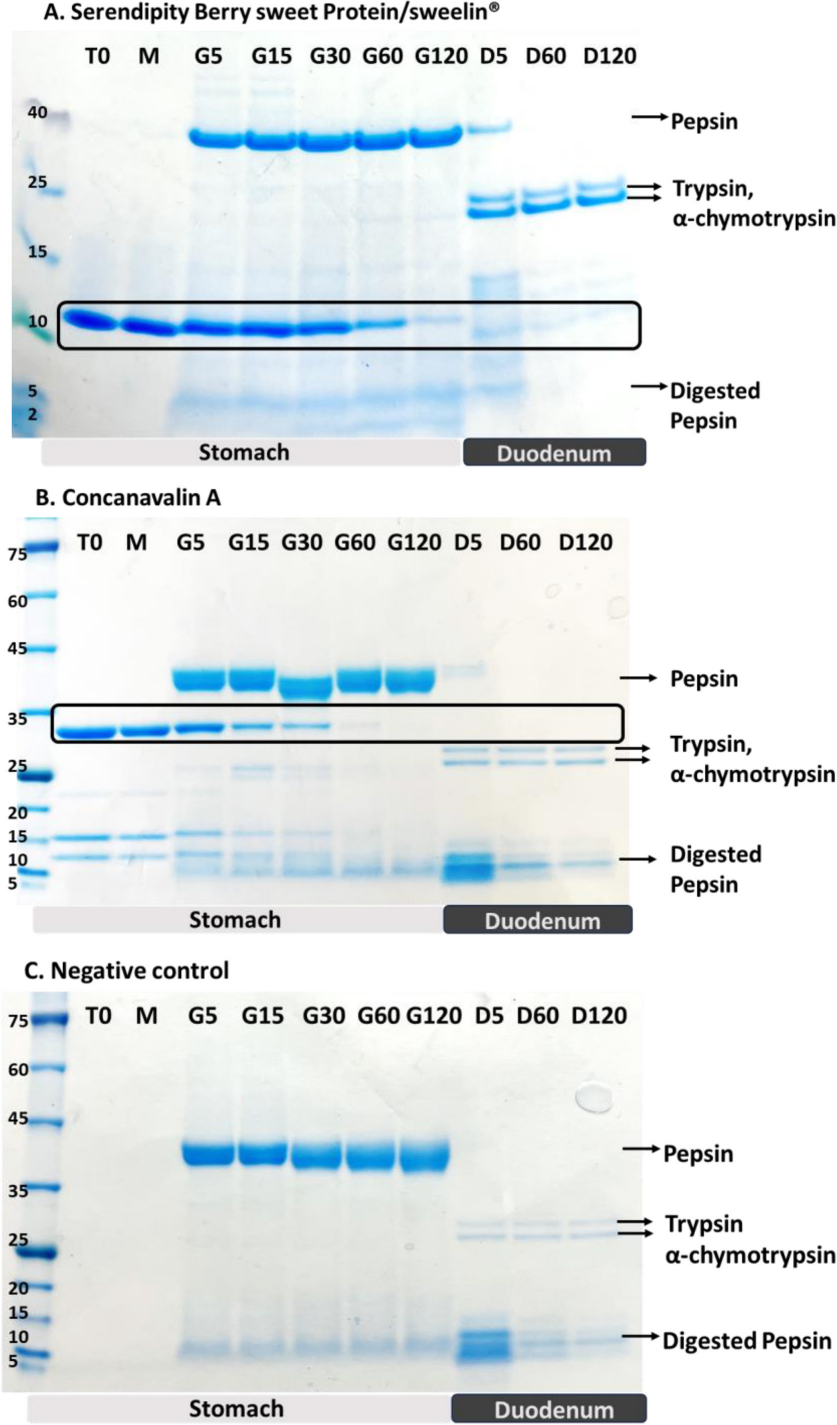
sweelin digestion pattern using semi‐dynamic model. Coomassie blue stain SDS‐PAGE gels of the digestion pattern of 20‐mg protein before digestion (TO), at oral phase (M), and gastric phase (G) following 5, 15, 30, 60 and 120 min of digestion, and at duodenal phase (marked as D) following 5, 60 and 120 min. Protein digesta samples were loaded on Tricine SDS‐PAGE gel (10%–20%) allowing better resolution of the migration of lower molecular weight proteins, whereas the controls were loaded on bis‐tris SDS‐PAGE gels. (A) sweelin digestion. (B) sweelin digestion pattern was compared with the digestion of positive control of jack bean protein, Concanavalin A (jack beans). (C) Negative control without protein in the present of digestive enzymes. Alt text: SDS‐PAGE gels demonstrating the digestion of sweelin, a control protein (Concanavalin A) and a negative control overtime in a simulated digestion model.

**FIGURE 5:**
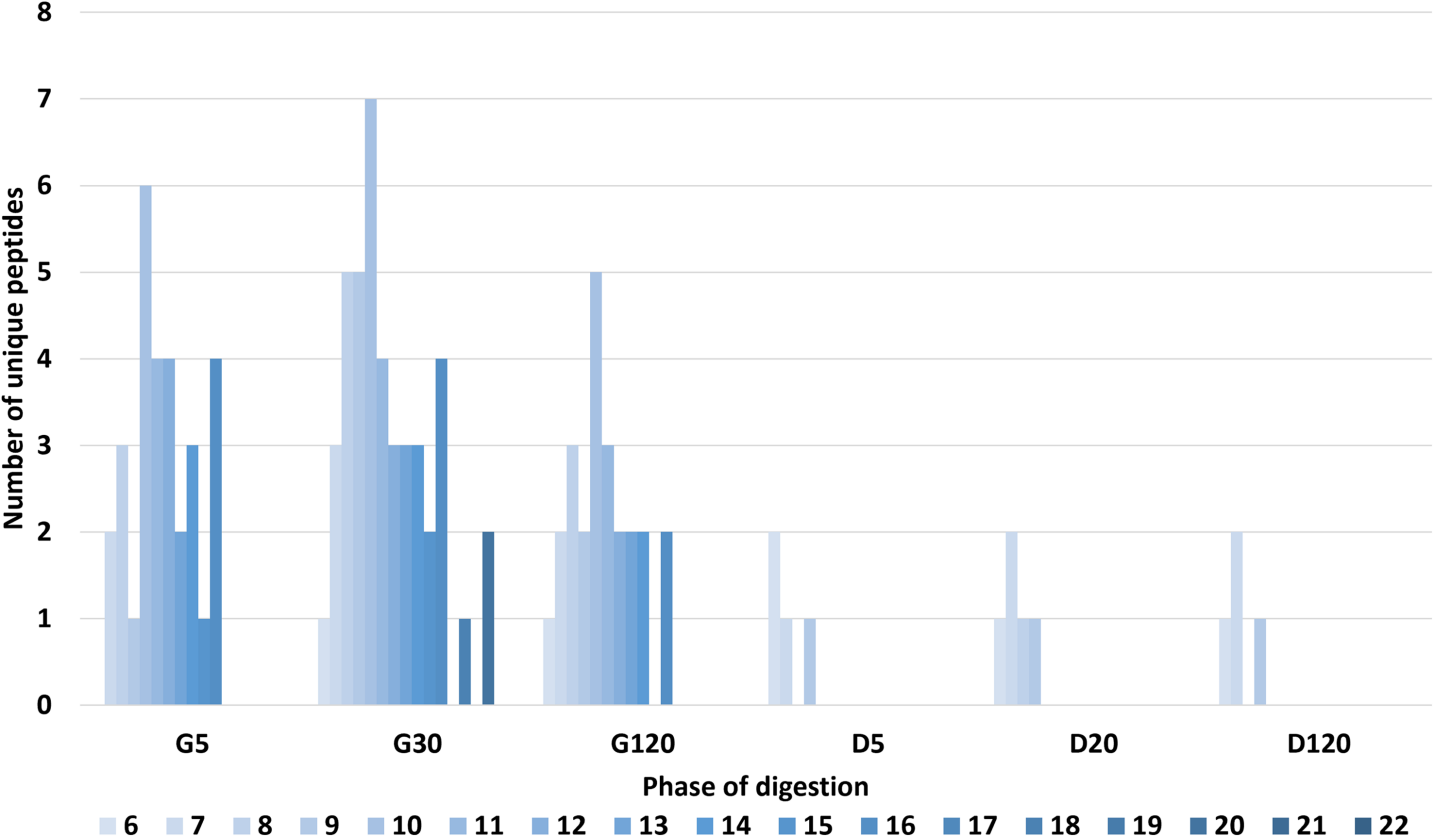
sweelin peptides formed in the semi‐dynamic digestion model. Peptide length distribution of 20‐mg protein analysed by LC–MS/MS. Peptides ranging in length from 6 to 22 amino acids (indicated by colour intensity gradient) were identified in the gastric phase (G) after 5, 30 and 120 min and in the duodenal phase (D) after 5, 60 and 120 min. Alt text: Graph demonstrating the resulting peptides from sweelin digestion after various timepoints.

The Coomassie Brilliant Blue–stained SDS‐PAGE gels identified the digestion pattern of the 20 mg of sweelin (DM31) protein as MW bands of 11 kDa (Figure 4A). During the 120 min of the gastric phase, the bands gradually diminished in intensity, indicating progressive digestion, which was followed by the complete digestion in the duodenal phase (Figure 4A). sweelin digestion was compared with that of Concanavalin A (Con A), a plant lectin purified from jack beans that is common in the diet. Con A was used as a positive control and appeared as a MW band around 30 kDa (Figure 4B). Con A was fully digested only after 60 min in the gastric phase, similar to the sweelin digestion pattern in the gastric phase. Digestion of a sample with no protein added was used as the negative control (Figure 4C). The Coomassie blue–stained SDS‐PAGE gels also detected the digestion enzymes used in the assays. Pepsin, added during the gastric phase, appeared as a band around 40 kDa (Figure 4A–C) and was further digested once reaching the duodenal phase, forming a smear between 5 and 15 kDa. Trypsin and α‐chymotrypsin, which were added only to the duodenal phase, were identified as bands located at 20–25 kDa, respectively.

sweelin digesta samples were analysed by LC–MS/MS to identify the peptides that formed during the gastric and duodenal digestion phases (Figure 5). The minimum detectable peptide length was six amino acids, with a 10 kDa cut‐off to exclude larger proteins. The results showed that in accordance with the progress in digestion, there was a marked decrease in the number of longer peptides as the digestion moved from the gastric phase (‘G’ in Figure 5) to the duodenal phase (‘D’ in Figure 5).

### 3.3 Genotoxicity Studies

#### 3.3.1 Bacterial Reverse Mutation Test

sweelin showed neither mutagenic nor cytotoxic potential in the bacterial reverse mutation test (Table 1). The test article did not produce any biologically relevant increases in the number of revertant colonies in any of the *S. typhimurium* tester strains. All responses to treatment were found to be within historical control ranges. Positive controls verified the sensitivity of the assay and the metabolising activity of the S9 preparations, as they induced increases in mean revertant colony numbers of at least twice (or three times in the case of strains TA1535 and TA1537) that of the vehicle controls, with mean values well in excess of historical negative control ranges.

**TABLE 1:** Bacterial reverse mutation test conducted with sweelin.

Concentration (μg/plate)	Revertant colonies per plate (mean ± SD)									
	TA98		TA100		TA1535		TA1537		TA102	
	−S9	+S9	−S9	+S9	−S9	+S9	−S9	+S9	−S9	+S9
Plate incorporation assay										
0	42.0 ± 3.6	39.0 ± 13.7	166.0 ± 30.3	198.0 ± 22.6	15.7 ± 3.8	25.3 ± 2.5	10.7 ± 4.2	8.0 ± 2.6	315.7 ± 18.8	307.0 ± 24.4
5	38.3 ± 13.1	47.3 ± 9.1	188.3 ± 8.6	172.7 ± 9.1	12.0 ± 5.3	44.0 ± 14.5	10.7 ± 6.7	10.0 ± 1.0	309.7 ± 10.3	295.3 ± 8.4
16	44.7 ± 4.0	50.0 ± 17.3	185.7 ± 17.6	190.3 ± 17.6	18.3 ± 2.5	36.7 ± 12.1	12.7 ± 3.1	12.0 ± 4.0	322.0 ± 13.0	300.7 ± 10.4
50	33.7 ± 6.0	46.0 ± 2.0	170.0 ± 11.4	170.7 ± 4.2	12.0 ± 6.1	26.0 ± 12.5	10.7 ± 1.2	12.0 ± 3.0	300.3 ± 6.7	314.3 ± 11.8
160	36.0 ± 5.2	44.7 ± 7.4	195.0 ± 27.7	161.7 ± 8.6	16.0 ± 3.0	42.7 ± 14.2	8.7 ± 4.6	8.3 ± 0.6	302.3 ± 9.1	327.0 ± 11.8
500	33.7 ± 3.2	42.7 ± 5.5	185.3 ± 33.8	182.3 ± 18.7	17.7 ± 3.1	34.3 ± 5.5	19.0 ± 8.7	13.0 ± 4.6	291.7 ± 15.9	328.7 ± 11.7
1600	39.7 ± 0.6	44.3 ± 3.5	167.7 ± 6.1	176.7 ± 6.8	13.3 ± 2.5	38.3 ± 17.6	11.3 ± 3.1	9.0 ± 4.4	309.0 ± 14.4	332.3 ± 15.5
5000	30.7 ± 1.5	55.0 ± 6.1	148.3 ± 18.5	192.7 ± 24.0	12.7 ± 3.2	25.3 ± 11.2	13.0 ± 0.0	7.7 ± 4.7	315.3 ± 22.5	335.7 ± 6.7
Pos (−S9)^a^	125.3 ± 49.5	NA	1036.3 ± 95.4	NA	663.3 ± 26.2	NA	90.3 ± 22.0	NA	936.0 ± 98.1	NA
Pos (+S9)^b^	NA	257.7 ± 43.8	NA	2155 ± 310.1	NA	343.7 ± 6.4	NA	58.0 ± 3.6	NA	655.3 ± 12.0
Preincubation assay										
0 (Neg)	25.7 ± 1.2	36.3 ± 4.5	137.0 ± 4.6	166.0 ± 9.6	14.3 ± 2.9	19.0 ± 10.8	11.7 ± 3.1	16.3 ± 9.0	312.0 ± 24.3	350.3 ± 21.2
50	22.7 ± 1.5	35.0 ± 5.2	141.0 ± 20.8	143.3 ± 14.5	15.7 ± 2.9	17.3 ± 9.5	12.3 ± 6.7	20.0 ± 4.6	343.3 ± 19.1	347.0 ± 33.2
160	23.3 ± 5.1	32.7 ± 6.0	132.7 ± 27.6	139.7 ± 8.1	13.3 ± 3.1	21.3 ± 4.7	16.3 ± 4.9	13.7 ± 3.1	329.7 ± 15.9	331.3 ± 11.0
500	21.0 ± 1.7	31.0 ± 7.9	127.7 ± 30.2	136.0 ± 21.9	11.0 ± 2.6	22.3 ± 0.6	15.7 ± 8.5	16.0 ± 3.5	324.0 ± 16.5	354.0 ± 43.3
1600	23.0 ± 2.6	28.7 ± 0.6	140.0 ± 23.5	148.3 ± 18.9	15.3 ± 8.1	17.0 ± 10.4	14.0 ± 3.6	17.0 ± 5.0	314.0 ± 31.8	332.7 ± 5.5
5000	27.3 ± 3.5	31.7 ± 4.5	119.3 ± 5.7	160.7 ± 22.0	16.0 ± 4.6	14.7 ± 6.0	16.0 ± 1.7	20.0 ± 10.8	307.0 ± 45.2	351.3 ± 17.6
Pos (−S9)^a^	174.7 ± 3.5	NA	629.0 ± 29.6	NA	618.7 ± 34.5	NA	340.7 ± 108.3	NA	856.3 ± 42.8	NA
Pos (+S9)^b^	NA	93.0 ± 8.9	NA	2405.7 ± 102.8	NA	332.0 ± 37.0	NA	64.3 ± 2.3	NA	612.3 ± 25.0

Abbreviations: +S9 = with metabolic activation; −S9 = without metabolic activation; 2‐AA = 2‐aminoanthracene; 2‐NF = 2‐nitrofluorene; 9‐AA = 9‐aminoacridine; B[a]P = benzo[a]pyrene; CHP = cumene hydroperoxide; Danthron = 1,8‐dihydroxyanthraquinone; NA = not applicable; NaN_3_ = sodium azide; Neg = negative; Pos = positive; SD = standard deviation.

^a^ Positive controls +S9: TA98 = 5 μg/plate B[a]P; TA100 and TA1535 = 5 μg/plate 2‐AA; TA1537 = 5 μg/plate B[a]P; TA102 = 30 μg/plate Danthron.

^b^ Positive controls −S9: TA98 = 2 μg/plate 2‐NF; TA100 and TA1535 = 2 μg/plate NaN_3_; TA1537 = 50 μg/plate 9‐AA; TA102 = 100 μg/plate CHP.

#### 3.3.2 *In Vitro* Mammalian Micronucleus Assay

sweelin did not induce excessive cytotoxicity (cytostasis ≤ 18% in initial toxicity assessment; results not shown); hence, the maximum concentration tested was 2000 μg/mL, the highest recommended by OECD Test Guideline 487 (*In Vitro Mammalian Cell Micronucleus Test*—OECD 2023). sweelin did not show any evidence of clastogenic or aneugenic activity in human peripheral blood lymphocytes in the short‐term assay with metabolic activation and the continuous assay (Table 2). In the short‐term assay without metabolic activation, a statistically significant increase (*p* < 0.05) in the proportion of micronucleated cells was observed at 2000 μg/mL (results not shown). The increases fell within the 95th percentile historical control ranges for the vehicle control. As the results did not fulfil the criteria for a clearly negative result, an additional 1000 binucleate cells per culture were evaluated to determine the frequency of micronuclei in the short‐term assay without metabolic activation. The results from the additional analyses demonstrated that the frequency of micronuclei in the test cultures was not significantly different from those observed in the concurrent vehicle controls (Table 2). These results fulfilled the criteria for a clearly negative result.

**TABLE 2:** *In vitro* mammalian cell micronucleus test in human peripheral blood lymphocytes treated with sweelin.

Concentration (μg/mL)	CPBI/% cytotoxicity^a^	Micronucleus cells scored (Cultures 1, 2 and total)^b^	Micronucleated cell frequency (Cultures 1, 2 and mean %)
3‐h treatment: −S9			
Negative control (culture medium)	1.69/NA	7, 5, 6, 9, 27	0.35, 0.25, 0.30, 0.45, 0.34
500	1.71/0	7, 7, 14	0.35, 0.35, 0.35
1000	1.78/0	8, 9, 17	0.40, 0.45, 0.43
2000	1.73/0	7, 10, 17	0.35, 0.50, 0.43
Positive controls:			
MMC (0.30 μg/mL)	1.42/39	34, 33, 67	1.70, 1.65, 1.68^c^, ^d^
COL (0.07 μg/mL)	1.51/26	29, 30, 59	1.45, 1.50; 1.48^c^, ^d^
3‐h treatment: +S9			
Negative control (culture medium)	1.76/NA	4, 3, 2, 2, 11	0.40, 0.30, 0.20, 0.20, 0.28
500	1.77/0	2, 2, 4	0.20, 0.20, 0.20
1000	1.78/0	2, 4, 6	0.20, 0.40, 0.30
2000	1.71/7	3, 3, 6	0.30, 0.30, 0.30
Positive control:			
CPA (10 μg/mL)	1.41/46	12, 12, 24	1.20, 1.20, 1.20^c^, ^d^
20‐h continuous treatment: −S9			
Negative control (culture medium)	1.77/NA	2, 5, 2, 2, 11	0.20, 0.50, 0.20, 0.20, 0.28
500	1.80/0	2, 3, 5	0.20, 0.30, 0.25
1000	1.78/0	4, 4, 8	0.40, 0.40, 0.40
2000	1.73/5	2, 3, 5	0.20, 0.30, 0.25
Positive controls:			
MMC (0.10 μg/mL)	1.62/20	18, 18, 36	1.80, 1.80, 1.88^c^, ^d^
COL (0.02 μg/mL)	1.55/28	12, 12, 24	1.20, 1.20, 1.20^c^, ^d^

Abbreviations: +S9 = with metabolic activation; −S9 = without metabolic activation; CBPI = cytokinesis‐block proliferation index; COL = colchicine; CPA = cyclophosphamide; MMC = mitomycin C; NA = not applicable.

^a^ Relative to negative control.

^b^ The negative control had four replicate cultures of 1000 scored cells each.

^c^ *p* < 0.001, *t*‐test.

^d^ Numbers exceeded negative control range.

The test as a whole was considered to be valid, as all conditions of validity were met: concurrent negative/vehicle controls were consistent with laboratory historical controls; concurrent positive controls induced a statistically significant increase in micronuclei and within normative ranges for each respective positive control; and a minimum of 50% of cells had gone through at least one cell division (as measured by binucleate + multinucleate cell counts) in vehicle control cultures at the time of harvest.

### 3.4 Repeated Dose Dietary Toxicity Studies in Rodents

#### 3.4.1 14‐Day Dietary Study

At the end of the 14‐day dosing period, there were no mortalities or significant clinical observations. No significant differences in body weight, body weight gain, food consumption, food efficiency or macroscopic observations attributable to the dietary administration of sweelin were observed. The mean overall daily intake (Days 0–14) of the test article in rats fed target dietary concentrations of 3000, 6000 and 13,200 ppm was calculated to be 281.7, 607.2 and 1249.5 mg/kg body weight/day for males and 277.4, 604.2 and 1274.5 mg/kg body weight/day for females.

Based on the toxicological endpoints evaluated under the conditions of the 14‐day dietary study, there were no test article–related changes observed in any animal. It was concluded that sweelin is well tolerated and does not elicit any toxic adverse effects at dietary concentrations of up to 13,200 ppm (equivalent to a mean intake of 1249.5 and 1274.5 mg/kg body weight/day for males and females, respectively).

#### 3.4.2 90‐Day Dietary Study

All animals survived until scheduled necropsy following the 91‐day dosing period. No notable clinical signs related to the test article were observed. Alopecia of the forelimbs was observed in two of 10 mid‐dose males, one of 10 females in the control group and three of 10 females in the low‐dose group. These occurrences were incidental and were not considered to be toxicologically relevant. Ophthalmological examinations were normal for all animals. Test article–related changes were not observed in any treatment group in comparison with the control group for FOB tests, motor activity, body weight, body weight gain, food consumption and food efficiency. Over the 91‐day dosing period, the mean daily intakes of sweelin for test groups provided 3250, 6500 and 14,300 ppm sweelin in the diet were calculated to be 194.2, 388.0 and 838.3 mg/kg body weight/day, respectively, for males and 225.9, 448.1 and 946.0 mg/kg body weight/day, respectively, for females. In the study, the targeted nominal intakes of approximately 250, 500 and 1100 mg/kg body weight/day were achieved.

Overall, there were no indications of any adverse effects of sweelin on any of the measured parameters (Tables 3 and 4). There were minor statistically significant changes observed in the haematological, coagulation, clinical chemistry and thyroid hormone levels. Monocytes were decreased in high‐dose females, and mean corpuscular haemoglobin concentration (MCHC) was decreased in mid‐dose females. APTT was increased in mid‐ and high‐dose females. No changes in coagulation parameters were observed in treated males. Clinical chemistry evaluations revealed an increase in serum glucose in low‐dose males and a decrease in serum calcium in high‐dose females. No significant changes were reported for urinalysis parameters in any treatment group. Upon thyroid hormone analysis, increases in TSH were observed in mid‐ and high‐dose males and females, along with an increase in T4 in mid‐dose females.

**TABLE 3:** Haematology values for male and female rats administered with sweelin in the diet for 90 days.

Parameter measured (mean ± SD)	Dose group (mg/kg body weight/day)							
	Males (*n* = 10)				Females (*n* = 10)			
	0 (control)^a^	250	500	1100	0 (control)^a^	250	500	1100
ABAS (×10^3^/μL)	0.15 ± 0.08	0.16 ± 0.08	0.18 ± 0.11	0.15 ± 0.06	0.07 ± 0.02	0.08 ± 0.04	0.05 ± 0.03	0.11 ± 0.06
AEOS (×10^3^/μL)	0.15 ± 0.05	0.17 ± 0.08	0.27 ± 0.36	0.16 ± 0.05	0.11 ± 0.038	0.11 ± 0.03	0.09 ± 0.02	0.10 ± 0.04
ALUC (×10^3^/μL)	0.15 ± 0.08	0.11 ± 0.031	0.12 ± 0.063	0.098 ± 0.04	0.054 ± 0.022	0.06 ± 0.02	0.041 ± 0.01	0.045 ± 0.03
ALYM (×10^3^/μL)	8.15 ± 1.41	7.72 ± 1.30	7.50 ± 1.96	7.67 ± 2.31	4.95 ± 1.60	4.52 ± 1.05	4.06 ± 0.93	4.33 ± 1.95
AMON (×10^3^/μL)	0.33 ± 0.06	0.37 ± 0.15	0.41 ± 0.26	0.31 ± 0.10	0.18 ± 0.07	0.16 ± 0.04	0.12 ± 0.05	0.12 ^b^ ± 0.05
ANEU (×10^3^/μL)	1.57 ± 0.42	1.79 ± 1.20	2.24 ± 2.17	1.68 ± 0.34	0.80 ± 0.40	0.73 ± 0.21	0.80 ± 0.36	0.71 ± 0.24
ARET (×10^3^/μL)	220.29 ± 55.35	213.35 ± 39.72	202.81 ± 39.72	220.27 ± 45.57	143.24 ± 38.54	151.51 ± 25.14	150.25 ± 37.10	143.94 ± 27.39
HCT (%)	47.66 ± 1.78	48.72 ± 1.62	48.25 ± 2.01	48.22 ± 1.36	46.18 ± 2.19	47.54 ± 1.66	46.69 ± 1.61	47.95 ± 1.97
HGB (g/dL)	15.32 ± 0.59	15.53 ± 0.55	15.44 ± 0.67	15.55 ± 0.49	15.64 ± 0.72	15.70 ± 1.21	15.13 ± 1.14	15.98 ± 0.77
MCV (fL)	55.09 ± 1.50	55.83 ± 1.37	55.84 ± 1.33	54.95 ± 1.11	56.39 ± 0.85	56.47 ± 1.35	56.15 ± 1.26	56.72 ± 1.34
MCH (pg)	17.67 ± 0.68	17.88 ± 0.57	17.89 ± 0.50	17.70 ± 0.42	19.09 ± 0.51	18.93 ± 0.58	18.67 ± 0.50	18.89 ± 0.28
MCHC (g/dL)	32.05 ± 0.56	31.99 ± 0.45	32.04 ± 0.35	32.21 ± 0.34	33.86 ± 0.49	33.55 ± 0.57	33.21 ± 0.44	33.34 ± 0.46
PLT (×10^3^/μL)	1053.7 ± 114.1	1013.7 ± 136.9	1054.0 ± 137.3	962.5 ± 106.1	907.0 ± 107.0	915.3 ± 93.9	953.2 ± 104.4	874.5 ± 219.1
RBC (×10^6^/μL)	8.67 ± 0.52	8.73 ± 0.33	8.64 ± 0.36	8.78 ± 0.29	8.20 ± 0.46	8.42 ± 0.32	8.32 ± 0.35	8.46 ± 0.44
RDW (%)	13.35 ± 1.37	12.88 ± 1.18	12.99 ± 0.80	13.07 ± 0.79	11.15 ± 0.64	11.21 ± 0.44	10.99 ± 0.55	11.11 ± 0.26
WBC (×10^3^/μL)	10.51 ± 1.69	10.30 ± 1.87	10.72 ± 3.84	10.08 ± 2.66	6.16 ± 1.78	5.67 ± 1.06	5.16 ± 1.27	5.41 ± 2.20

Abbreviations: ABAS = absolute basophils; AEOS = absolute eosinophils; ALUC = absolute leukocytes; ALYM = absolute lymphocytes; AMON = absolute monocytes; ANEU = absolute neutrophils; ARET = absolute reticulocytes; HCT = haematocrit; HGB = haemoglobin; MCH = mean corpuscular haemoglobin; MCHC = mean corpuscular haemoglobin concentration; MCV = mean corpuscular volume; PLT = platelet count; RBC = red blood cell; RDW = red blood cell distribution width; SD = standard deviation; WBC = white blood cell.

^a^ Control animals were administered water.

^b^ Significantly different from control (Anova & Dunnett test, *p* ≤ 0.05).

**TABLE 4:** Clinical chemistry, coagulation, thyroid hormone and urinalysis values for male and female rats administered with sweelin in the diet for 90 days.

Parameter measured (mean ± SD)	Dose group (mg/kg body weight/day)							
	Males (*n* = 10)				Females (*n* = 10)			
	0 (control)^a^	250	500	1100	0 (control)^a^	250	500	1100
Clinical chemistry								
ALT (U/L)	32.8 ± 18.0	37.6 ± 22.4	34.8 ± 28.3	35.2 ± 10.1	38.0 ± 47.4	25.9 ± 7.4	26.5 ± 5.9	19.4 ± 3.8
ALB (g/dL)	3.92 ± 0.26	3.99 ± 0.19	3.82 ± 0.24	3.97 ± 0.09	4.46 ± 0.37	4.56 ± 0.51	4.42 ± 0.38	4.31 ± 0.46
ALKP (U/L)	76.6 ± 14.2	71.3 ± 16.2	66.9 ± 11.7	83.5 ± 11.0	36.1 ± 10.5	34.1 ± 9.8	38.2 ± 13.2	34.5 ± 9.0
AST (U/L)	75.5 ± 18.9	85.3 ± 29.9	79.5 ± 30.6	82.1 ± 14.2	105.2 ± 78.2	81.6 ± 16.6	92.1 ± 30.5	71.5 ± 16.2
CALC (mg/dL)	10.70 ± 0.64	10.98 ± 0.56	10.37 ± 0.44	10.54 ± 0.55	10.40 ± 0.47	10.01 ± 0.77	9.73 ± 0.60	9.60* ± 0.71
CL (mmol/L)	97.6 ± 1.11	97.7 ± 0.92	98.50 ± 2.01	97.02 ± 1.31	97.0 ± 2.49	96.48 ± 3.22	94.78 ± 2.30	94.40 ± 2.04
CHOL (mg/dL)	65.3 ± 16.0	82.0 ± 17.6	67.5 ± 13.3	68.6 ± 11.2	76.3 ± 18.0	79.9 ± 16.8	79.0 ± 13.4	77.5 ± 19.8
CREA (mg/dL)	0.26 ± 0.03	0.27 ± 0.02	0.24 ± 0.04	0.28 ± 0.03	0.31 ± 0.06	0.27 ± 0.04	0.28 ± 0.04	0.26 ± 0.08
GGT (U/L)	1.50 ± 0.00	1.50^b^ ± 0.00	1.50^b^ ± 0.00	1.50^b^ ± 0.00	1.50 ± 0.00	1.50^b^ ± 0.00	1.50^b^ ± 0.00	1.50^b^ ± 0.00
GLOB (g/dL)	2.34 ± 0.16	2.42 ± 0.21	2.30 ± 0.31	2.36 ± 0.12	2.01 ± 0.37	1.87 ± 0.25	1.83 ± 0.31	1.69 ± 0.21
GLUC (mg/dL)	209.2 ± 27.1	246.0* ± 28.0	197.6 ± 23.3	234.3 ± 36.2	176.7 ± 25.1	178.5 ± 25.1	187.0 ± 33.9	188.5 ± 33.8
HDL (mmol/L)	1.03 ± 0.26	1.33 ± 0.36	1.08 ± 0.25	1.13 ± 0.20	1.56 ± 0.38	1.57 ± 0.34	1.59 ± 0.22	1.54 ± 0.38
IPHS (mg/dL)	8.62 ± 1.09	8.78 ± 0.84	8.07 ± 0.99	8.68 ± 0.78	8.11 ± 1.34	7.76 ± 0.95	7.60 ± 1.44	7.10 ± 1.22
LDL (mmol/L)	0.27 ± 0.10	0.33 ± 0.10	0.28 ± 0.10	0.27 ± 0.09	0.16 ± 0.05	0.17 ± 0.05	0.16 ± 0.06	0.17 ± 0.05
K (mmol/L)	6.98 ± 0.88	7.06 ± 1.07	6.85 ± 0.77	7.34 ± 1.14	7.61 ± 2.01	6.61 ± 1.45	6.34 ± 2.20	5.91 ± 1.35
NA (mmol/L)	140.0 ± 1.3	141.1 ± 1.3	140.4 ± 2.1	139.9 ± 0.9	137.1 ± 3.6	136.5 ± 3.5	135.1 ± 2.8	134.3 ± 3.6
SDH (U/L)	18.74 ± 10.95	25.99 ± 17.57	19.19 ± 20.48	17.51 ± 6.75	18.22 ± 27.66	12.75 ± 4.53	11.40 ± 6.27	10.46 ± 2.21
BILI (mg/dL)	0.08 ± 0.03	0.08 ± 0.03	0.07 ± 0.02	0.05 ± 0.01	0.05 ± 0.02	0.06 ± 0.02	0.07 ± 0.03	0.04 ± 0.03
TP (g/dL)	6.26 ± 0.39	6.41 ± 0.33	6.12 ± 0.42	6.33 ± 0.11	6.47 ± 0.51	6.43 ± 0.68	6.25 ± 0.39	6.00 ± 0.56
TRIG (mg/dL)	81.4 ± 23.9	98.4 ± 32.9	93.6 ± 50.9	89.5 ± 34.5	54.0 ± 18.6	62.3 ± 33.8	73.3 ± 34.7	60.5 ± 15.8
BUN (mg/dL)	15.7 ± 2.1	15.9 ± 1.0	16.8 ± 1.7	16.2 ± 2.7	17.7 ± 2.5	16.5 ± 2.8	16.2 ± 2.9	18.3 ± 2.7
Coagulation								
APTT (s)	18.26 ± 1.27	18.00 ± 1.83	18.48 ± 1.43	18.32 ± 1.62	14.17 ± 1.08	15.27 ± 0.78	17.38^##^ ± 2.30	16.23** ± 1.51
PT (s)	10.15 ± 0.26	10.05 ± 0.30	10.23 ± 0.19	10.34 ± 0.33	9.66 ± 0.17	9.60 ± 0.31	9.69 ± 0.53	9.92 ± 0.28
Thyroid hormone								
TSH (ng/mL)	4.41 ± 0.33	4.53 ± 0.18	4.91^##^ ± 0.47	5.18^###^ ± 0.41	4.32 ± 0.31	4.56 ± 0.17	4.60^+^ ± 0.19	5.14^+++^ ± 0.59
T4 (ng/mL)	44.31 ± 2.43	41.96 ± 2.51	44.74 ± 2.66	41.55 ± 3.32	38.78 ± 2.82	37.39 ± 2.41	43.14** ± 3.45	38.78 ± 2.13
T3 (ng/mL)	1.49 ± 0.31	1.73 ± 0.25	1.48 ± 0.42	1.38 ± 0.27	2.08 ± 0.59	2.47 ± 0.84	3.14 ± 1.36	2.06 ± 1.01
Urinalysis								
Urine volume (mL)	8.55 ± 3.35	7.40 ± 4.22	5.65 ± 2.89	5.10 ± 2.5	3.56 ± 3.98^c^	3.56 ± 2.72^c^	2.59 ± 2.82	7.33 ± 5.17^c^
pH	6.55 ± 0.50	6.50 ± 0.41	6.50 ± 0.41	6.55 ± 0.44	6.56 ± 1.01^c^	6.44 ± 0.53^c^	6.70 ± 0.75	6.39 ± 0.55^c^
Urine glucose (mg/dL)	0.0 ± 0.0	10.0 ± 31.6	10.0 ± 31.6	0.0 ± 0.0	33.3 ± 50.0^c^	22.2 ± 44.1^c^	0.0 ± 0.0	33.3 ± 50.0^c^
Urine ketone (mmol/L)	3.0 ± 2.6	4.0 ± 2.1	4.5 ± 1.6	7.5 ± 5.4	2.2 ± 5.1^c^	0.6 ± 1.7^c^	3.5 ± 6.3	0.0 ± 0.0^c^
Urine protein (mg/dL)	24.0 ± 10.5	43.5 ± 40.1	76.5 ± 85.8	58.0 ± 36.1	132.2 ± 130.1^c^	52.8 ± 46.0^c^	147.5 ± 135.2	36.7 ± 48.5^c^
Specific gravity	1.03 ± 0.006	1.03 ± 0.004	1.03 ± 0.003	1.03 ± 0.002	1.03 ± 0.006^c^	1.03 ± 0.004^c^	1.03 ± 0.008	0.96 ± 0.17^c^
Urobilinogen (EU/dL)	0.20 ± 0.00	0.28 ± 0.25	0.36 ± 0.34	0.36 ± 0.34	0.76 ± 0.61^c^	0.47 ± 0.40^c^	0.88 ± 0.70	0.47 ± 0.40^c^

*Note:* Significantly different from control (Anova & Dunnett test, *p ≤ 0.05; **p ≤ 0.01). Significantly different from control (Anova & Dunnett test [Log], ^##^ *p* ≤ 0.01; ^###^ *p* ≤ 0.001). Significantly different from control (Anova & Dunnett test [Rank], ^+^ *p* ≤ 0.05; ^+++^ *p* ≤ 0.001).

Abbreviations: ALB = albumin; ALKP = alkaline phosphatase; ALT = alanine aminotransferase; APTT = activated partial thromboplastin time; AST = aspartate aminotransferase; BILI = bilirubin; BUN = blood urea nitrogen; CALC = calcium; CHOL = cholesterol; CL = chloride; CREA = creatine; EU = Ehrlich units; GGT = gamma‐glutamyl transferase; GLOB = globulin; GLUC = glucose; HDL = high‐density lipoprotein; IPHS = inorganic phosphorous; K = potassium; LDL = low‐density lipoprotein; NA = sodium; PT = prothrombin time; s = seconds; SD = standard deviation; SDH = sorbitol dehydrogenase; T3 = triiodothyronine; T4 = thyroxine; TP = total protein; TRIG = triglycerides; TSH = thyroid‐stimulating hormone.

^a^ Control animals were administered water.

^b^ Inappropriate for statistics (Anova & Dunnett rank test).

^c^ *n* = 9 were used in this group for the statistical analysis.

Following necropsy, no test article–related macroscopic or microscopic findings were observed. Fluid‐filled uteruses were observed in females in the low‐dose group (three of 10), mid‐dose group (one of 10) and high‐dose group (two of 10). All microscopic observations were interpreted as incidental findings based on their low frequency, random distribution across treatment groups or an expected occurrence in rats of this strain and age. No test article–related changes in absolute or relative organ weights were observed in any treatment group (Tables 5 and 6).

**TABLE 5:** Absolute and relative organ weights of male and female rats administered with sweelin in the diet for 90 days.

Parameter measured (mean ± SD)	Dose group (mg/kg body weight/day)							
	Males (*n* = 10)				Females (*n* = 10)			
	0 (control)^a^	250	500	1100	0 (control)^a^	250	500	1100
Absolute organ weights								
Terminal body weight (g)	557.8 ± 36.0	572.7 ± 34.1	579.4 ± 58.4	552.4 ± 32.5	300.4 ± 32.1	297.0 ± 28.4	297.9 ± 31.1	309.6 ± 34.7
Adrenal glands (g)	0.06 ± 0.01	0.07 ± 0.01^b^	0.068 ± 0.01	0.07 ± 0.005	0.07 ± 0.01	0.07 ± 0.009	0.08 ± 0.01	0.07 ± 0.007
Brain (g)^c^	2.20 ± 0.11	2.25 ± 0.08	2.25 ± 0.07	2.29 ± 0.10	1.94 ± 0.06	1.93 ± 0.12	1.98 ± 0.083	2.00 ± 0.05
Epididymides (g)	1.58 ± 0.10	1.60 ± 0.16	1.64 ± 0.12	1.69 ± 0.15	—	—	—	—
Heart (g)	1.44 ± 0.14	1.55 ± 0.13	1.50 ± 0.20	1.42 ± 0.08	0.93 ± 0.09	0.99 ± 0.09	0.96 ± 0.08	0.97 ± 0.05
Kidneys (g)	3.14 ± 0.14	3.33 ± 0.30	3.20 ± 0.33	3.20 ± 0.21	1.91 ± 0.24	1.91 ± 0.15	1.88 ± 0.15	1.93 ± 0.15
Liver (g)	13.13 ± 1.41	14.55 ± 1.43	13.77 ± 2.52	13.49 ± 1.33	7.77 ± 1.21	7.75 ± 0.92	8.06 ± 0.93	7.79 ± 0.88
Ovaries with oviducts (g)	—	—	—	—	0.13 ± 0.01	0.13 ± 0.03	0.13 ± 0.02	0.14 ± 0.02
Pituitary gland (g)	0.017 ± 0.002	0.015 ± 0.003	0.018 ± 0.004	0.016 ± 0.002	0.021 ± 0.003	0.02 ± 0.003^b^	0.02 ± 0.003	0.02 ± 0.003
Pro, SV, CG combined (g)	3.74 ± 0.30	3.73 ± 0.39	4.08 ± 0.66	4.01 ± 0.45	—	—	—	—
Spleen (g)	0.88 ± 0.12	0.89 ± 0.10	0.93 ± 0.11	0.80 ± 0.08	0.53 ± 0.09	0.52 ± 0.08	0.51 ± 0.04	0.51 ± 0.08
Testes (g)^d^	3.84 ± 0.19	3.83 ± 0.30	3.73 ± 0.51	3.928 ± 0.283	—	—	—	—
Thymus (g)^d^	0.32 ± 0.08	0.32 ± 0.03	0.28 ± 0.08	0.31 ± 0.07	0.29 ± 0.07	0.27 ± 0.09	0.25 ± 0.06	0.26 ± 0.07
Thyroid‐parathyroid (g)^d^	0.03 ± 0.01	0.03 ± 0.004	0.03 ± 0.004	0.03 ± 0.005	0.027 ± 0.005	0.03 ± 0.003	0.03 ± 0.007	0.03 ± 0.006
Uterus (g)	—	—	—	—	0.68 ± 0.24	0.72 ± 0.23	0.68 ± 0.14	0.77 ± 0.3
Relative organ‐brain weight ratio (%)								
Adrenal glands (g)	0.03 ± 0.005	0.030 ± 0.005^b^	0.03 ± 0.005	0.031 ± 0.003	0.037 ± 0.007	0.038 ± 0.005	0.038 ± 0.007	0.035 ± 0.004
Epididymides (g)	0.72 ± 0.06	0.71 ± 0.069	0.73 ± 0.04	0.74 ± 0.06	—	—	—	—
Heart (g)	0.65 ± 0.065	0.69 ± 0.063	0.66 ± 0.08	0.62 ± 0.031	0.48 ± 0.05	0.52 ± 0.064	0.48 ± 0.04	0.48 ± 0.04
Kidneys (g)	1.43 ± 0.078	1.48 ± 0.12	1.42 ± 0.15	1.40 ± 0.12	0.98 ± 0.13	1.00 ± 0.12	0.95 ± 0.08	0.96 ± 0.07
Liver (g)^c^	5.97 ± 0.64	6.47 ± 0.68	6.11 ± 1.0	5.89 ± 0.48	4.00 ± 0.59	4.03 ± 0.54	4.08 ± 0.45	3.89 ± 0.41
Ovaries with oviducts(g)	—	—	—	—	0.069 ± 0.006	0.068 ± 0.013	0.068 ± 0.01	0.07 ± 0.009
Pituitary (g)^d^	7.67 ± 0.60	6.79 ± 1.34	8.02 ± 1.49	7.11 ± 0.73	10.9 ± 1.32	10.3 ± 1.80^b^	10.22 ± 1.61	11.2 ± 1.68
Pro, SV, CG (g)	1.70 ± 0.17	1.66 ± 0.18	1.81 ± 0.27	1.76 ± 0.23	—	—	—	—
Spleen (g)	0.40 ± 0.04	0.40 ± 0.04	0.41 ± 0.05	0.35^e^ ± 0.03	0.27 ± 0.05	0.27 ± 0.04	0.26 ± 0.02	0.26 ± 0.04
Testes (g)^d^	1.75 ± 0.082	1.70 ± 0.15	1.66 ± 0.244	1.72 ± 0.12	—	—	—	—
Thymus (g)	0.14 ± 0.04	0.14 ± 0.014	0.13 ± 0.04	0.13 ± 0.03	0.15 ± 0.04	0.14 ± 0.05	0.13 ± 0.03	0.13 ± 0.03
Thyroid‐parathyroid (g)^d^	0.013 ± 0.005	0.012 ± 0.002	0.013 ± 0.002	0.013 ± 0.002	0.014 ± 0.002	0.014 ± 0.002	0.0167 ± 0.004	0.015 ± 0.003
Uterus (g)	—	—	—	—	0.35 ± 0.13	0.38 ± 0.13	0.34 ± 0.07	0.39 ± 0.15
Relative organ‐body weight ratio (%)								
Adrenal glands (g)	0.11 ± 0.01	0.12 ± 0.02^b^	0.12 ± 0.02	0.13 ± 0.01	0.24 ± 0.06	0.25 ± 0.04	0.26 ± 0.07	0.23 ± 0.04
Brain	3.96 ± 0.24	3.94 ± 0.29	3.92 ± 0.38	4.15 ± 0.15	6.53 ± 0.72	6.56 ± 0.87	6.70 ± 0.75	6.52 ± 0.64
Epididymides (g)	2.84 ± 0.26	2.79 ± 0.26	2.84 ± 0.26	3.06 ± 0.19	—	—	—	—
Heart (g)	2.58 ± 0.15	2.71 ± 0.14	2.58 ± 0.18	2.58 ± 0.12	3.12 ± 0.28	3.37 ± 0.34	3.22 ± 0.25	3.15 ± 0.37
Kidneys (g)	5.65 ± 0.28	5.81 ± 0.47	5.53 ± 0.52	5.81 ± 0.43	6.35 ± 0.51	6.46 ± 0.42	6.35 ± 0.60	6.26 ± 0.52
Liver (g)	23.52 ± 1.64	25.38 ± 1.60	23.67 ± 2.56	24.40 ± 1.62	25.81 ± 2.27	26.13 ± 2.27	27.11 ± 2.31	25.18 ± 1.62
Ovaries with oviducts (g)	—	—	—	—	0.45 ± 0.05	0.44 ± 0.08	0.46 ± 0.07	0.46 ± 0.07
Pituitary (g)	0.003 ± 0.0003	0.003 ± 0.0006	0.003 ± 0.0007	0.003 ± 0.0003	0.007 ± 0.0006	0.007 ± 0.001^b^	0.007 ± 0.001	0.007 ± 0.001
Pro, SV, CG (g)	0.007 ± 0.001	0.007 ± 0.001	0.007 ± 0.001	0.007 ± 0.001	—	—	—	—
Spleen (g)	1.58 ± 0.17	1.56 ± 0.16	1.61 ± 0.15	1.45 ± 0.12	1.78 ± 0.31	1.74 ± 0.20	1.72 ± 0.18	1.66 ± 0.18
Testes (g)	6.91 ± 0.53	6.71 ± 0.69	6.48 ± 0.95	7.12 ± 0.48	—	—	—	—
Thymus (g)^d^	0.57 ± 0.13	0.55 ± 0.055	0.50 ± 0.18	0.56 ± 0.13	0.96 ± 0.23	0.89 ± 0.29	0.85 ± 0.14	0.840 ± 0.24
Thyroid‐Parathyroid (g)^c^, ^d^	0.51 ± 0.21	0.46 ± 0.073	0.50 ± 0.09	0.53 ± 0.08	0.91 ± 0.23	0.91 ± 0.04	1.11^f^ ± 0.28	0.10 ± 0.12
Uterus (g)	—	—	—	—	2.31 ± 0.86	2.48 ± 0.88	2.30 ± 0.52	2.54 ± 1.11

Abbreviations: — = not applicable; CG = coagulating gland; Pro = prostate; SD = standard deviation; SV = seminal vesicles.

^a^ Control animals were administered water.

^b^ *n* = 9 were used in this group for the statistical analysis.

^c^ Non‐parametric analysis in females (Kruskal–Wallis & Dunn).

^d^ Non‐parametric analysis in males (Kruskal–Wallis & Dunn).

^e^ Significantly different from control (Anova & Dunnett test, *p* ≤ 0.05).

^f^ Significantly different from control (Kruskal–Wallis & Dunn, *p* ≤ 0.05).

**TABLE 6:** Terminal body weight and organ weights of male and female rats administered sweelin in the diet for 90 days.

Parameter measured (mean ± SD)	Dose group (mg/kg body weight/day)							
	Males (*n* = 10)				Females (*n* = 10)			
	0 (control)^a^	250	500	1000	0 (control)^a^	250	500	1000
Body weight (g)	557.8 ± 36.0	572.7 ± 34.1	579.4 ± 58.4	552.4 ± 32.5	300.4 ± 32.1	297.0 ± 28.4	297.9 ± 31.1	309.6 ± 34.7
Adrenal glands (g)	0.06 ± 0.01	0.07 ± 0.01^b^	0.07 ± 0.01	0.07 ± 0.005	0.072 ± 0.013	0.072 ± 0.009	0.075 ± 0.01	0.070 ± 0.007
Brain (g)^c^	2.2 ± 0.1	2.25 ± 0.08	2.3 ± 0.07	2.29 ± 0.09	1.94 ± 0.06	1.93 ± 0.12	1.98 ± 0.08	2.00 ± 0.05
Epididymides (g)	1.66 ± 0.095	1.66 ± 0.16	1.64 ± 0.12	1.69 ± 0.15	—	—	—	—
Heart (g)	1.44 ± 0.1	1.5 ± 0.13	0.15 ± 0.21	1.42 ± 0.08	0.93 ± 0.09	1.00 ± 0.09	0.96 ± 0.08	0.97 ± 0.05
Kidneys (g)	3.1 ± 0.1	3.33 ± 0.30	3.20 ± 0.33	3.20 ± 0.21	1.91 ± 0.24	1.91 ± 0.15	1.88 ± 0.15	1.93 ± 0.15
Liver (g)	13.1 ± 1.4	14.55 ± 1.43	13.77 ± 2.52	13.49 ± 1.33	7.77 ± 1.21	7.75 ± 0.92	8.06 ± 0.93	7.79 ± 0.88
Ovaries with oviducts (g)	—	—	—	—	0.13 ± 0.01	0.13 ± 0.03	0.13 ± 0.02	0.14 ± 0.017
Pituitary gland (g)	0.017 ± 0.002	0.015 ± 0.003	0.018 ± 0.004	0.016 ± 0.002	0.02 ± 0.003	0.02 ± 0.003	0.02 ± 0.003	0.02 ± 0.003
Pro, SV, CG (combined) (g)	3.74 ± 0.30	3.73 ± 0.39	4.1 ± 0.7	4.00 ± 0.45	—	—	—	—
Spleen (g)	0.99 ± 0.12	0.89 ± 0.10	0.9 ± 1.1	0.80 ± 0.08	0.53 ± 0.09	0.52 ± 0.08	0.51 ± 0.04	0.51 ± 0.08
Testes (g)	3.83 ± 0.19	3.83 ± 0.3	3.73 ± 0.5	3.93 ± 0.28	—	—	—	—
Thymus (g)	0.32 ± 0.078	0.32 ± 0.026	0.3 ± 0.08	0.31 ± 0.072	0.29 ± 0.070	0.27 ± 0.09	0.25 ± 0.056	0.3 ± 0.07
Thyroid‐Parathyroid (g)	0.028 ± 0.01	0.026 ± 0.004	0.03 ± 0.004	0.029 ± 0.005	0.026 ± 0.005	0.027 ± 0.003	0.033 ± 0.007	0.03 ± 0.006
Uterus (g)	—	—	—	—	0.68 ± 0.24	0.72 ± 0.23	0.68 ± 0.14	0.8 ± 0.3

Abbreviations: — = not applicable; CG = coagulating gland; Pro = prostate; SD = standard deviation; SV = seminal vesicles.

^a^ Control animals were administered water.

^b^ *n* = 9 were used in this group for the statistical analysis.

^c^ Non‐parametric analysis in females (Kruskal–Wallis & Dunn).

Under the conditions of the 90‐day dietary study, the no‐observed‐adverse‐effect level (NOAEL) for sweelin administered in the diet was determined to be 14,300 ppm in Sprague Dawley rats, which is equivalent to a dietary intake of 838.3 and 946.0 mg sweelin/kg body weight/day in male and female rats, respectively.

## 4 Discussion

In order to support the safety of Amai Proteins' highly purified novel sweet protein ingredient, Serendipity Sweet Protein (sweelin), produced via precision fermentation, a battery of *in vitro* and *in vivo* studies was conducted, including standard *in vitro* reverse mutation and mammalian micronucleus assays, as well as a 90‐day dietary toxicity study in rats.

Amai Proteins investigated the interaction and activation of the sweet taste receptors T1R2/T1R3 by sweelin (Figure 3). It is reasonable to conclude that the loss of the sweet taste after 135 s indicates that sweelin ceases to activate the sweet taste receptor beyond this timepoint. This suggests that the protein does not have prolonged effects on the sweet taste receptor and thus may not be associated with a risk of desensitisation or downregulation of the sweet taste receptor.

The digestibility of the protein was evaluated in an *in vitro* semi‐dynamic digestion model based on the INFOGEST protocol (Brodkorb et al. 2019; Shani‐Levi et al. 2013). Adapting a pharmacological approach, a standardised *in vitro* digestion model was selected, mirroring the physiological conditions of healthy adult gastro‐duodenal digestion, aimed to consolidate a standardised protocol for simulated digestion of foods that are close to the physiological condition. The results showed that partly digested sweelin, after gastric acidic and enzymatic degradation, was readily digestible under intestinal conditions where bile and pancreatic secretions were present. In fact, the presence of physiological levels of trypsin and α‐chymotrypsin led to the degradation of over 95% of sweelin. LC–MS/MS analysis of bioaccessible peptides formed in the gastric and duodenal phases reaffirmed these observations that sweelin is readily digested in the intestine into very short peptides. After 2 h of intestinal digestion, undigested protein levels were estimated to be less than 3% of the total ingested dose. The results of the *in vitro* digestibility study suggest that the digestion of sweelin is similar to that of other proteins commonly found in food. Therefore, following ingestion, it is expected that sweelin would be effectively digested into small peptides and amino acids in the small intestine by digestion enzymes. The results of the digestibility study suggest that sweelin has a low risk for allergenicity, as the protein is readily digested into small peptides that are not expected to present a cross‐reactivity potential to known allergens. Furthermore, the low risk for allergenicity of sweelin was evaluated using bioinformatic methods, including prediction with artificial intelligence. No significant sequence homology matches were identified to putative allergens that shared greater than 35% identity over a sliding window of 80 amino acids or any exact matches over eight amino acids, indicating that sweelin would be unlikely to have any allergenic cross‐reactivity according to the guidelines recommended by FAO/WHO (2001) and Codex Alimentarius (2003, 2009). This conclusion aligns with the findings presented by Freeman et al. (2024), who used similar bioinformatic methods to demonstrate that their single‐chain monellin shows no indicators of potential allergenicity.

sweelin's lack of genotoxic potential was demonstrated in the two *in vitro* genotoxicity studies. sweelin was not genotoxic in the *in vitro* reverse mutation assay, both in the presence and absence of metabolic activation by the S9 mix. In the short‐term micronucleus assay without metabolic activation, a significant increase (0.45%) in the mean micronucleated cells was observed at 2000 μg/mL, although the value fell within the 95th percentile historical vehicle control range (0.2%–1.0%). Upon analysing an additional 1000 binucleate cells per culture, this result was not reproducible, and no positive responses were observed. Based on the results of the micronucleus study, sweelin does not have clastogenic or aneugenic potential.

No adverse effects related to the dietary administration of sweelin were observed in the 90‐day repeated dose oral toxicity study at up to 14,300 ppm, the highest dose tested. The few statistically significant findings observed during the study period were not toxicologically relevant, as they were not associated with histopathological correlates or other biologically relevant endpoints, they did not follow a dose–response relationship, values were within the historical control ranges, and the findings were present in only one sex. Although an increase in TSH was observed in both males and females in the mid‐ and high‐dose groups, all values were within the historical control range and there were no correlating changes in the levels of T3 or T4, or adverse microscopic findings in the thyroid or pituitary glands. Additionally, there were no statistically significant organ weight differences (absolute/relative to body and brain weight) for either the thyroid or pituitary. Therefore, the changes in TSH levels were not considered to be related to the dietary administration of sweelin. The fluid‐filled uteruses observed in some of the female animals were considered a normal finding consistent with the rodent oestrous cycle in animals of this strain and age (as shown by the frequency in the control group) and, therefore, not considered to be test article–related.

Based on the lack of any toxicologically relevant findings, the NOAEL for sweelin was established as 14,300 ppm, which is equivalent to a dietary intake of 838.3 and 946.0 mg sweelin/kg body weight/day in male and female rats, respectively.

The safety of sweelin is also supported by a clinical trial conducted in Sourasky Medical Center (Tel Aviv, Israel). Twenty healthy volunteers that were enrolled to the study consumed a single dose of 51 mg of sweelin aligned to the sweetness equivalent of 75 g of glucose, as used in a standard oral glucose tolerance test (OGTT) (unpublished study). All participants completed the study with no test article–related adverse events, and there were no dropouts. Additional data related to sweelin clinical study are being prepared for publication and will be detailed in a separate manuscript.

Furthermore, potential side effects were evaluated by the aforementioned sensory panel. Thirty‐two panellists completed a structured questionnaire based on the validated General Assessment of Side Effects (GASE) questionnaire (Rief et al. 2011). Participants were asked to report any side effects experienced during or after sensory evaluation sessions and rate their severity. The majority of responses (84.3%) indicated ‘not present’ or ‘mild’ for all side effects, with no reports of severe side effects. Notably, no health issues or adverse effects were reported by any panel member throughout the evaluation period.

sweelin is a novel hyper‐sweet protein, and to the authors' knowledge, no toxicological studies of the active constituent of sweelin (the DM31 sweet protein) are available in the literature. Safety evaluations for single‐chain monellin proteins derived from *K. phaffii* have been conducted by Novik et al. (2023) and Freeman et al. (2024). Novik et al. (2023) evaluated the safety of a single‐chain monellin. In the bacterial reverse mutation test (OECD Test Guideline 471, *Bacterial Reverse Mutation Test*—OECD 1997), monellin was tested in *S. typhimurium* strains TA100, TA98 and TA97 at up to 80 mg/mL with or without metabolic activation. No mutagenic responses were reported, which is consistent with the findings for sweelin. The investigators also conducted a subchronic and chronic study in guinea pigs and rats, respectively (Novik et al. 2023). The studies were GLP compliant, but adherence to specific study guidelines was not indicated. In the subchronic study, guinea pigs (*n* = 9/sex/group, ages 2–2.5 months) were administered 1.45 or 14.5 mg monellin/kg body weight/day by gavage for 21 days. No deaths or abnormal clinical signs were reported, and no adverse findings were revealed upon clinical chemistry analysis and histopathological evaluations. The chronic study was conducted in rats (*n* = 10/sex/group) administered 1.43 or 14.3 mg monellin/kg body weight/day by gavage for 150 days. The endpoints assessed were similar to those prescribed under OECD Test Guideline 408 (*Repeated Dose 90‐Day Oral Toxicity Study in Rodents*—OECD 1998b). After the 150‐day dosing period, no test article–related findings were reported for rats in either test group. Although the doses tested in these studies were much lower than those tested for sweelin, the studies corroborate the safety of monellin in guinea pigs and in rats for longer dosing periods.

Single‐chain monellin protein (MNEI) is composed of monellin Chains A and B linked together by a glycine–phenylalanine residues (Gly‐Phe). Freeman et al. (2024) evaluated a single‐chain monellin referred to as serendipity berry sweet protein (SBSP). MON, the active constituent of SBSP, shares 92.7% sequence homology to DM31 that characterises Amai Proteins' sweelin. Amai Proteins' DM31 is a novel single‐chain monellin redesigned by AI‐CPD resulting in improved protein stability with a better sensory profile compared with single‐chain monellin as assessed by professional sensory panels.

Similar to the current evaluation, the Freeman et al. (2024) protein was evaluated in an *in vitro* protein digestion assay, *in vitro* genotoxicity studies and a 13‐week oral dietary study in rats. All studies showed similar findings as reported in the current study. In the 13‐week dietary study conducted in rats receiving up to 2000 mg SBSP/kg body weight/day, no treatment‐related effects were reported. The authors established the NOAEL as 2000 mg/kg body weight/day, the highest dose tested, which corresponds to 1954 and 1967 mg/kg body weight/day in males and females, respectively. When expressed on the basis of monellin content (20.9%) in SBSP, 1954 mg/kg body weight/day corresponds to 408 mg/kg body weight/day demonstrating lower purity of the single chain monellin in the final product compared with sweelin. Freeman et al. (2024) further calculated an acceptable daily intake of 4.1 mg/kg body weight/day for monellin in SBSP using an uncertainty factor of 100.

## 5 Conclusions

Amai Proteins' Serendipity Berry Sweet Protein, or sweelin, was evaluated in an *in vitro* digestibility study and a series of standard toxicological studies, including a bacterial reverse mutation test, an *in vitro* mammalian cell micronucleus assay and a 90‐day repeated dose dietary toxicity study in rats. The results of the studies demonstrate that sweelin is digested in the gastric phase, does not have any genotoxic potential and does not have any adverse effects in rats provided up to 14,300 ppm sweelin in the diet for 91 days. Accordingly, the NOAEL was determined to be 14,300 ppm, the highest dose tested, which is equivalent to a dietary intake of 838.3 and 946.0 mg sweelin/kg body weight/day in male and female rats, respectively.

Overall, the studies conducted with Amai Proteins' Serendipity Berry Sweet Protein, or sweelin, support the safe use of the novel sweet protein as a sweetening agent in food and beverage products.

## Author Contributions

**Yael Lifshitz:** conceptualisation, project administration, supervision, data curation, investigation, resources, methodology, validation, visualisation and drafting and critically revising the manuscript. **Shira Paz:** conceptualisation, data curation, investigation, methodology, validation, visualisation and drafting and critically revising the manuscript. **Rotem Saban:** conceptualisation, data curation, investigation, methodology, validation, visualisation and drafting and critically revising the manuscript. **Inbar Zuker:** data curation, formal analysis, investigation and methodology. **Hagay Shmuely:** data curation, methodology and investigation. **Katy Gorshkov:** data curation and investigation. **Jwar Meetro:** drafting and data curation. **Shahrzad Tafazoli:** conceptualisation, resources and drafting and critically revising the manuscript. **Trung Vo:** drafting and data curation. **Gabriela Amiram:** investigation. **Carmit Shani Levi:** conceptualisation, investigation, methodology and resources. **Uri Lesmes:** methodology and project administration. **Ilan Samish:** funding acquisition, project administration, conceptualization, supervision, resources and critically revising the manuscript.

## Conflicts of Interest

Jwar Meetro, Shahrzad Tafazoli and Trung Vo are employees of Intertek Health Sciences Inc., which has provided consultancy services to Amai Proteins Ltd. Yael Lifshitz, Shira Paz, Rotem Saban, Inbar Zuker, Hagay Shmuely, Katy Gorshkov and Ilan Samish are employees of Amai Proteins Ltd.

## Data Availability

The data that support the findings of this study are available from the corresponding author upon reasonable request.
